# Exhaustive Analysis of a Genotype Space Comprising 10^15^ Central Carbon Metabolisms Reveals an Organization Conducive to Metabolic Innovation

**DOI:** 10.1371/journal.pcbi.1004329

**Published:** 2015-08-07

**Authors:** Sayed-Rzgar Hosseini, Aditya Barve, Andreas Wagner

**Affiliations:** 1 Institute of Evolutionary Biology and Environmental Sciences, University of Zürich, Zürich, Switzerland; 2 Swiss Institute of Bioinformatics, Quartier Sorge, Batiment Genopode, Lausanne, Switzerland; 3 Santa Fe Institute, Santa Fe, New Mexico, United States of America; Christian Albrechts Universität zu Kiel, GERMANY

## Abstract

All biological evolution takes place in a space of possible genotypes and their phenotypes. The structure of this space defines the evolutionary potential and limitations of an evolving system. Metabolism is one of the most ancient and fundamental evolving systems, sustaining life by extracting energy from extracellular nutrients. Here we study metabolism’s potential for innovation by analyzing an exhaustive genotype-phenotype map for a space of 10^15^ metabolisms that encodes all possible subsets of 51 reactions in central carbon metabolism. Using flux balance analysis, we predict the viability of these metabolisms on 10 different carbon sources which give rise to 1024 potential metabolic phenotypes. Although viable metabolisms with any one phenotype comprise a tiny fraction of genotype space, their absolute numbers exceed 10^9^ for some phenotypes. Metabolisms with any one phenotype typically form a single network of genotypes that extends far or all the way through metabolic genotype space, where any two genotypes can be reached from each other through a series of single reaction changes. The minimal distance of genotype networks associated with different phenotypes is small, such that one can reach metabolisms with novel phenotypes – viable on new carbon sources – through one or few genotypic changes. Exceptions to these principles exist for those metabolisms whose complexity (number of reactions) is close to the minimum needed for viability. Increasing metabolic complexity enhances the potential for both evolutionary conservation and evolutionary innovation.

## Author Summary

Genotype-phenotype mapping is one of the ultimate goals of computational systems biology, and can provide new insights into the function and evolution of biological systems. We present a comprehensive genotype-phenotype map for a space of metabolic genotypes that comprises more than 10^15^ central carbon metabolisms. Only one in a million of these metabolisms can sustain life on any one of 10 carbon sources we consider, but these viable metabolisms form connected genotype networks that extend far through genotype space. In addition, they render multiple novel metabolic phenotypes in their immediate neighborhood accessible through small evolutionary changes that require only the alteration of single metabolic reactions. The map we construct reveals an organization of core metabolism that simultaneously facilitates evolutionary conservation of existing metabolic phenotypes, and the origination of novel metabolic traits that allow viability on novel carbon sources. Such metabolic innovation is essential, particularly for organisms that experience unexpected environmental changes, and that explore or invade new habitats.

## Introduction

Attempts to understand the relationship between genotype and phenotype have played a pivotal role in the history of genetics and evolutionary biology, beginning with the rediscoveries of Mendel’s laws, which revealed genotypes as distinct from phenotypes and responsible for the inheritance of phenotypic traits [1]. Subsequent efforts to map individual genes encoding such traits onto chromosomes helped develop classical and molecular genetic mapping methods [2]. Decades later, genome sequencing facilitated more systematic studies of the relationship between genotypes and complex traits, and resulted in the emergence of functional genomics and systems biology. Today, a central goal of systems biology is to uncover and predict the relationship between genotypes and complex phenotypes, which include the structure and dynamics of complex intracellular networks like gene regulatory, signaling, and metabolic networks [3,4].

Computational methods to predict phenotype from genotype are increasingly powerful and they can help establish genotype-phenotype maps at unprecedented resolution [5–7]. An ideal such map would cover all possible genotypes, but unfortunately, the entire space of a system’s genotypes is usually too vast to study. For instance, the space of all RNA sequences of length 100 comprises 4^100^ = 10^60^ different RNA sequences, and the space of all protein sequences of length 100 comprises 20^100^ = 10^130^ different protein sequences. Exhaustive genotype-phenotype mapping using computational approaches is possible only in small systems. They include short hydrophobic polar (HP) model proteins folding on square and cubic lattices [8], short RNA sequences folding into planar secondary structures [9], and small gene regulatory networks [10]. Similar exhaustive approaches have not been taken yet in metabolic systems, and our understanding of metabolic systems is thus far limited to sampling of metabolic genotype space [11–18]. Here, we build on our previous work studying the connectivity of the space of central carbon metabolisms [19] and present an exhaustive genotype-phenotype map of this space.

In evolution, phenotypic change is caused by genotypic change. Thus, the structure of a genotype-phenotype map contains information about the evolutionary potential and limitations of an evolving system. Metabolisms are important classes of evolving systems, because they are a source of many evolutionary adaptions and innovations, especially in microorganisms. For instance, microorganisms have acquired the ability to utilize many non-natural substances like polychlorinated biphenyls, chlorobenzens, organic solvents and synthetic pesticides as food [20–23]. Microbial isolates from pristine soils can survive on several antibiotics like ciprofloxacin by using them as sole carbon sources [24], and halophilic bacteria can tolerate high salt concentration by synthesizing novel molecules like ectoine or glycine betaine [25,26].

An organism’s metabolism is a biochemical reaction network that comprises a set of reactions that are catalyzed by enzymes encoded by genes. Following common practice in the computational analysis of metabolism [4], we here represent a metabolic genotype as a binary presence-absence pattern of a set of biochemical reactions, i.e., as a binary string whose entries indicate presence (1) or absence (0) of a reaction among some set of *N* possible reactions. In our analysis (see also [19]), this global reaction set comprises *51* reactions from central carbon metabolism (see Methods, and S9 Table). Any one metabolism can be viewed as a point in metabolic genotype space whose size is equal to 2^51^ = 2.25 × 10^15^ metabolisms. In this framework, a metabolism’s genotype can evolve by either losing one or more reactions, for example through a loss of function mutation, or by gaining one or more reactions, for example through horizontal gene transfer or gain of function mutations [27–29].

We focus on central carbon metabolism because of its pivotal role in extracting energy from extracellular carbon sources. It comprises the interconnected biochemical pathways of glycolysis, gluconeogenesis, the pentose-phosphate pathway (PP), and the tricarboxylic acid cycle (TCA). These are supplemented by anaplerotic reactions and the glyoxylate shunt [30]. Glycolysis converts glucose into pyruvate and produces high-energy compounds like ATP and NADH. In parallel, the pentose-phosphate pathway generates NADPH and pentose sugars required for anabolic reactions. The glycolytic end product pyruvate is oxidized to acetyl-CoA, which enters the tri-carboxylic acid cycle (TCA), a cyclical series of reactions that generate ATP, NADH, and amino acid precursors. Pyruvate and phosphoenolpyruvate (PEP) can also enter the TCA cycle directly through anaplerotic reactions that replenish TCA cycle intermediates consumed in biosynthetic processes. Conversely, under gluconeogenic conditions, the TCA cycle intermediates oxaloacetate or malate are converted to pyruvate and PEP to provide the precursors for gluconeogenesis. Acetyl-CoA can also participate in the glyoxylate shunt to generate succinate for carbohydrate synthesis. Finally, reactions of the oxidative phosphorylation pathway participate in production of ATP from NADH (S1 Fig).

Not all organisms contain all reactions associated with central carbon metabolism. For example, although the Embden–Meyerhoff–Parnass (EMP) glycolytic pathway (S1 Fig) is nearly ubiquitous among eukaryotes, prokaryotes can use diverse natural glycolytic alternatives [31–33]. Archaebacteria like Sulfolobus solfataricus [34] and Thermoplasma acidophilum [35], metabolize glucose through the Entner-Doudoroff (ED) pathway and heterolactic fermentative bacteria use phosphoketolase pathway instead of the canonical glycolytic pathway [32]. Natural glycolytic pathways vary in their reaction content and in how much ATP they produce per glucose [33,36]. Furthermore, genome analysis of 19 species including 4 archaea, 14 bacteria and 1 eukaryote has revealed that in the majority of species the citric acid cycle is incomplete or absent [37]. Also, in vivo quantification of intracellular carbon fluxes from C_13_ tracer experiments has shown that relative activities of individual reactions of central carbon metabolism vary widely among different species [38]. Such variation calls for an examination of how genotypic variation maps into variation in metabolic phenotypes.

For our analysis, we define a metabolic phenotype based on a metabolism’s *viability*, its ability to sustain life in a given set of environments. We call a metabolism viable in any one environment if it can synthesize 13 key precursors (S1 Fig) that are produced by central carbon metabolism and that are necessary for the synthesis of amino acids and other essential biomass molecules [39]. We here consider 10 environments that vary in their carbon source and contain an otherwise minimal complement of nutrients (see Methods). We represent the phenotype of a given metabolism as a binary string of length 10 whose entries indicate viability (1) or inviability (0) of the metabolism on a given carbon source. In this framework, a change in metabolic genotype is a metabolic innovation if it leads to viability on new combinations of carbon sources. To compute the phenotype of a given metabolic genotype, we use the constraint-based method of flux balance analysis (FBA, see Methods) [7,40–42] whose qualitative predictions of viability are in good agreement with experimental data [43–49]. FBA takes advantage of constraints imposed by the stoichiometry of all metabolic reactions and maximal nutrient uptake rates in a given environment. Subject to the law of mass conservation in a metabolic steady state, it can predict the rate at which each reaction can proceed under conditions of maximal production of biomass precursors [7,40,44,47,50–52]. Although FBA is computationally efficient [7,40–42], determining the phenotypes of more than 10^15^ metabolic genotypes in each of 10 possible environments is challenging and required us to develop techniques that simplify this task (see Methods).

We use the resulting genotype-phenotype map to analyze a set of viable metabolisms with a given phenotype as a graph, where two nodes (metabolisms) are neighbors if they can be connected by a single reaction change. We study the organization of these graphs in metabolic genotype space, and find that they typically extend far through this space. To study a metabolism’s potential for metabolic innovation, that is, how changes in metabolic reactions can lead to new metabolic phenotypes (viability in new environments) we explore the metabolism’s neighborhood in genotype space and this neighborhood’s phenotypic diversity. We find that the neighborhoods of metabolisms with the same phenotype but different genotype often contain metabolisms with different novel metabolic phenotypes. Also, the genotype networks of different metabolic phenotypes abut each other in genotype space. Together, these observations suggest that the organization of metabolic genotype space is conducive to metabolic innovation.

## Results

### The number of viable metabolisms depends on carbon source and reaction numbers

**Fig 1:**
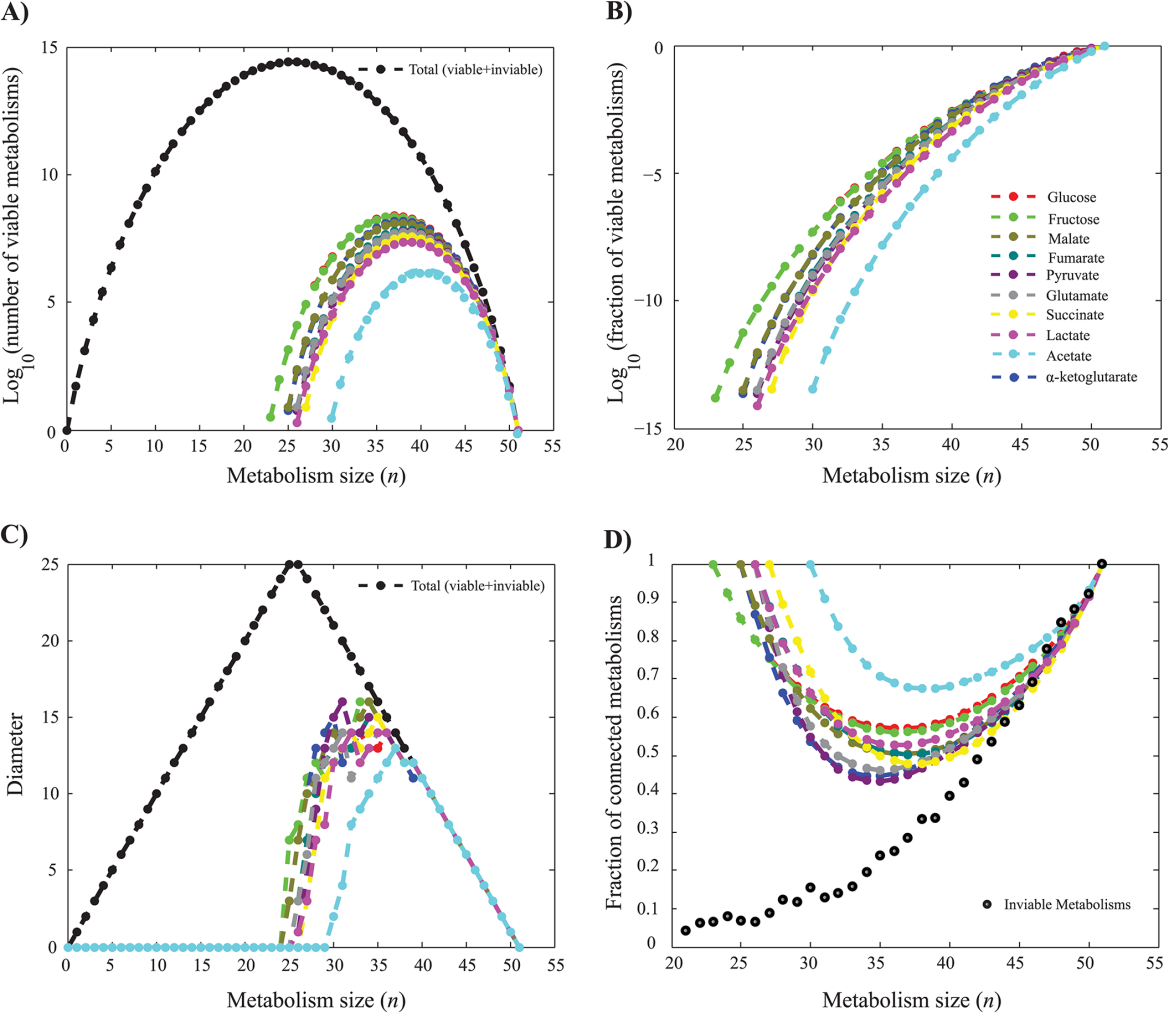
Metabolisms viable on different carbon sources and genotypic differences among them. **a)** Number of viable metabolisms. Black circles (vertical axis) indicate the total possible numbers (Nn) of metabolisms of a given size *n* (horizontal axis, *N* = 51, 0 ≤ *n* ≤ 51). Colored data points indicate the number of metabolisms viable on the single carbon sources indicated in the legend. **b)** Fraction of metabolisms viable on different carbon sources. Note the logarithmic vertical scale. Data on glucose in a) and b) has been previously published [19]. **c)** Genotype network diameter. Black circles indicate the diameter of genotype space for metabolisms of a given size, which is an upper bound to the diameter of any one genotype network. Colored data points indicate genotype network diameter for metabolisms viable on a single carbon source. Where sets of viable metabolisms comprised more than one connected component, the diameter of the giant component was chosen. At *n* ≥ 40, all genotype networks have the maximally possible diameter. **d)**. Fraction of metabolisms that contain no disconnected reactions. Colored circles correspond to metabolisms viable on the carbon source specified in the legend and black circles correspond to inviable metabolisms. Data on inviable metabolisms is based on 10000 randomly sampled inviable metabolisms. Interpolation between data points (circles) is linear and displayed as a visual guide.

We first enumerated the number of central carbon metabolisms (CCM) on a given carbon source. Fig 1A shows this number as a function of metabolism size, that is, the number *n* of reactions in a metabolism, for various carbon sources. Note the logarithmic vertical axis. Black data points indicate the total number of metabolisms of a given size *n*, regardless of their viability. This number is given by the binomial coefficient $\left(\begin{array}{c} N\\ n \end{array}\right)$. The following observations emerge from this figure. First, the minimum number of reactions in a viable metabolism is not the same for all carbon sources. For example, it varies from *n*_*min*_ = 23 for glucose and fructose to (*n*_*min*_ = 30) for acetate (S2 Fig, See the S1 Text for an explanation of this difference).

Second, the maximum number of viable metabolisms also varies by more than two orders of magnitude, from approximately 1.9×10^6^ for acetate to 2.4×10^8^ for glucose (see S1 Table).

Third, the number of viable metabolisms shows a unimodal distribution whose qualitative shape is predicted by binomial coefficients that are shifted by *n*_*min*_, i.e., (N−nminn−nmin), for any one carbon source (See S3A and S3B Fig for examples). This is a result of the fact that adding any number of reactions to a viable metabolism of minimal size *n*_*min*_ creates another viable metabolism, and there are (N−nminn−nmin) ways of adding *n* − *n*_*min*_ reactions. We note that this qualitative relationship is not quantitatively accurate, for reasons explained in the S2 Text.

Fig 1B shows the same information as Fig 1A, but expresses the number of viable metabolisms as a fraction of all metabolisms (Nn) at a given size *N*. Note the logarithmic scale and that the fraction of viable metabolisms declines faster than exponentially with decreasing *n*. It is easy to understand this pattern if one considers a metabolism (“the child”) that is derived from another (“the parent”) by eliminating a single reaction. First, some fraction of the children of viable parent metabolisms is inviable; second, this fraction increases as *n* decreases; third, all children of inviable metabolisms are themselves inviable. Together, these observations can account for the rapid decrease of the fraction of viable metabolisms.

### Except for the smallest metabolisms, most viable metabolisms form a genotype network with a large diameter

Viable genotypes of all but the smallest sizes *n* form a single connected network of genotypes, where any two genotypes can be reached from one another through changes in individual reactions. In a previous contribution, we have demonstrated this connectivity for metabolisms viable on glucose and on all ten carbon sources we consider here taken together [19]. S2 Table shows that it also holds for metabolisms viable on the nine other carbon sources. Even where the set of viable metabolisms is partitioned into more than one component (*n*_*C*_ > 1; S2 Table), i.e., more than one genotype network, this number of components is usually small, and the largest of them harbors a large fraction *r*_*G*_ of genotypes (*r*_*G*_ > 0.95 in 23 out of 29 cases where *r*_*G*_ < 1; S2 Table).

Starting from these observations, we asked how different the reaction content of metabolisms viable on the same carbon source could be. In graph theoretic terms, this is a question about the diameter of the set of viable genotypes at any one size *n*. Fig 1C shows this diameter as a function of metabolism size *n* for all 10 carbon sources. For most metabolism sizes, the diameter lies between 5 and 15, indicating that metabolisms of the same size and viable on the same carbon sources can have very different reaction complements. Moreover, for any one carbon source, and for metabolisms of most sizes above *n*_*min*_, the diameter as a fraction of the diameter of the genotype space (i.e. the maximum possible diameter (See methods)) is equal to one or very close to one (Fig 1C). This implies that the set of viable metabolisms is not highly localized within a small region of genotype space. Rather, its members occur throughout the space and the set comprising them often spans this space. Since the fraction of viable metabolisms increases with *n*, the fractional diameter increases with increasing *n* (until *n* = 40, where it reaches one for all carbon sources).

**Fig 2:**
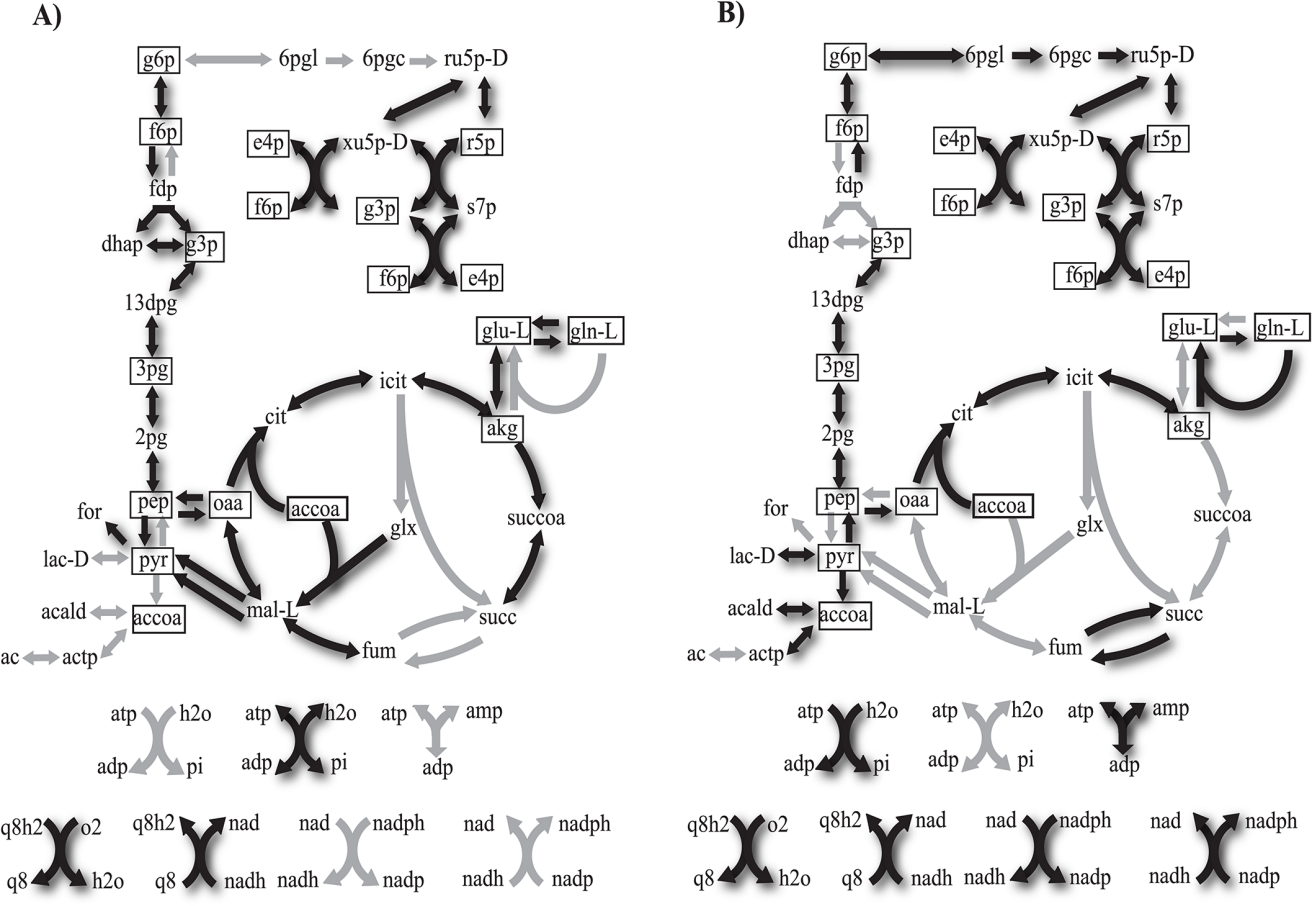
Example of two maximally different metabolisms. Each arrow in each panel corresponds to one of the 51 internal reactions we consider. Black arrows and gray arrows correspond to reactions that are present or absent, respectively, in the metabolism shown. Metabolites are indicated by their acronyms (see S9 Table). Boxed metabolites correspond to 13 essential biomass precursors. Note that 4 metabolites (accoa, g3p, f6p and e4p) are shown more than once for visual clarity. **a)** A metabolism with 33 reactions that is viable on fructose and that differs from another metabolism of size 33 viable on fructose shown in **b)** by 16 reactions. The two metabolisms share 17 reactions.

As an example of two viable networks comprising maximally different reaction sets, Fig 2. shows two maximally different metabolisms viable on fructose. Each metabolism contains 33 reactions. The two metabolisms share 17 reactions, and differ in 16 reactions. The products of the 17 shared reactions are the essential biomass precursors (boxed molecules in Fig 2.), meaning that the respective reactions cannot be bypassed via alternative reactions or pathways. In contrast, the 16 differing reactions are either non-essential reactions that do not directly contribute to the production of small biomass molecules, or they are reactions essential only in one of the metabolisms but not in both. Such non-shared but essential reactions exist, because a necessary biomass molecule may be synthesized by one or more alternative reactions or pathway in two metabolism. An example is the reaction catalyzed by glutaminase, which is essential for the synthesis of glutamate (glu-L) from glutamine (gln-L) and *α*-ketoglutarate (akg) in the metabolism of Fig 2B, but is substituted for by the reaction catalyzed by glutamate dehydrogenase, which is essential for production of glutamate from *α*-ketoglutarate (akg) in the metabolism of Fig 2A.

Central carbon metabolism contains pairs of reactions that differ only in the co-factor they use. Three such pairs are relevant for our analysis. The first comprises two reactions catalyzed by malic enzyme that use NAD and NADP as co-factors. The second comprises two reactions catalyzed by different transhydrogenases, and the third includes the reactions catalyzed by ATPase and ATP synthase (S9 Table). We wanted to examine whether such pairs of reactions trivially increase the genotypic distance among metabolism pairs and thus artificially inflate genotype network diameter. For any given genotype network, this could only be the case if the two members of all metabolism pairs with the highest genotypic distance use different reactions from at least one of these two pairs. To find out, we first enumerated all metabolism pairs with genotypic distance equal to the diameter of the given genotype network, and noted that there are up to millions of such pairs. Next, we examined for each such pair of metabolisms whether (i) one member used the NAD isoform of malic enzyme while the other one used the NADP isoform, or (ii) one member used one transhydrogenase while the other member used the other transhydrogenase or (iii) one member used ATPase and the other one ATP synthase. If so, we eliminated the pair from the set of pairs with largest distance. S3 Table shows the percentage of metabolism pairs that remained in the set based on this criterion. It exceeds 30% for most genotype networks and is greater than zero for every genotype network. This observation implies that in every genotype network at least one metabolism pair does not have artificially large genotypic distance due to co-factor dependency. Hence, co-factor dependency does not artificially inflate genotype network diameter.

Reactions that are blocked–they must have zero flux for stoichiometric reasons [11, 53]–could also contribute to large genotype network diameter. To find out whether this is the case, we calculated the total number of blocked reactions for each metabolism pair that is viable on the same carbon source and whose genotypic distance is equal to the diameter of its genotype network. S4 Table shows, for each carbon source, the minimum number of blocked reactions among such metabolism pairs. For most genotype networks (71.6% percent, 189 of 253) this minimum is zero. This means that at least one pair of metabolisms without any blocked reaction exists among the metabolism pairs with the largest genotypic distance. The exceptions are some genotype networks of metabolisms with intermediate sizes, where some contain no metabolism pairs without blocked reactions. Even in these genotype networks, however, only one or at most two reactions are blocked, meaning that the genotype network diameter would decrease only by this number if one were to disregard blocked reactions.

### The majority of reactions are connected to one another in most viable metabolisms

In a horizontal gene transfer event, enzyme-coding genes can be imported into a genome whose products catalyze reactions that may be connected to or disconnected from the resident metabolism. We define a reaction as disconnected from a metabolism if (i) its products are neither biomass precursors nor substrates of any other reaction in the resident metabolism, or (ii) at least one of its substrates is neither a product of other reactions nor a nutrient taken up from the environment. For example, among the 16 reactions that differ between the metabolisms in Fig 2., one reaction in each of the two metabolisms is disconnected from the rest, that is, its substrates are neither products of other reactions nor are they nutrients provided by the environment in which fructose is the sole carbon source. Specifically, in Fig 2A, the reaction that is catalyzed by malate synthase and produces L-malate (mal-L) from glyoxylate (glx) and acetyl-coenzyme A (accoa) is a disconnected reaction, because glyoxylate is neither available in the environment, nor is it produced by other reactions in the metabolism. Similarly, in Fig 2B the reaction that is catalyzed by fructose-bisphosphatase and produces fructose 6-phosphate (f6p) from fructose 1,6-bisphosphate (fdp) is disconnected.

To find out whether such disconnected metabolisms could strongly influence our analysis of metabolic genotype space, we determined how abundant they are. Specifically, we first computed the fraction *f*_*d*_ of all viable metabolisms that contained at least one such disconnected reaction. The value of *f*_*d*_ ranged from 0.517 for metabolisms viable on pyruvate to 0.307 for those viable on acetate. Fig 1D shows 1 − *f*_*d*_, i.e., the fraction of viable metabolisms containing only connected reactions as a function of metabolism size. We note that for any one carbon source, the smallest viable metabolisms contain only connected reactions. It is easy to see why this must be the case: if a minimal metabolism contained a disconnected reaction, then this reaction would by definition be dispensable, and its elimination could not abolish viability, which means that the metabolism could not possibly be minimal. Conversely, the largest viable metabolism contains all reactions we consider, which are also connected. Only at intermediate sizes do metabolisms with disconnected reactions occur. However, we also found that at most sizes, most viable metabolisms contain only connected reactions, regardless of the carbon source considered (Fig 1D). This stands in contrast to the fraction of *inviable* metabolisms lacking disconnected reactions (i.e. 1 − *f*_*d*_) (black circles in Fig 1D), which is much smaller for metabolisms up to about 42 reactions, where it approaches that of viable metabolisms.

Given these observation, it is thus of little surprise that the patterns we reported above extend to metabolisms where all reactions are connected. Specifically, the quasi-binomial dependence of the number of viable genotypes on *n*, and the greater than exponential reduction in the fraction of viable genotypes with decreasing *n*, are preserved (S4A and S4B Fig). Moreover, such metabolisms can also be quite different from one another (S4C Fig) and at most sizes *n*, most or all such metabolisms reside in a single connected genotype network (S5 Table).

### Metabolisms viable on multiple carbon sources show similar organization

**Fig 3:**
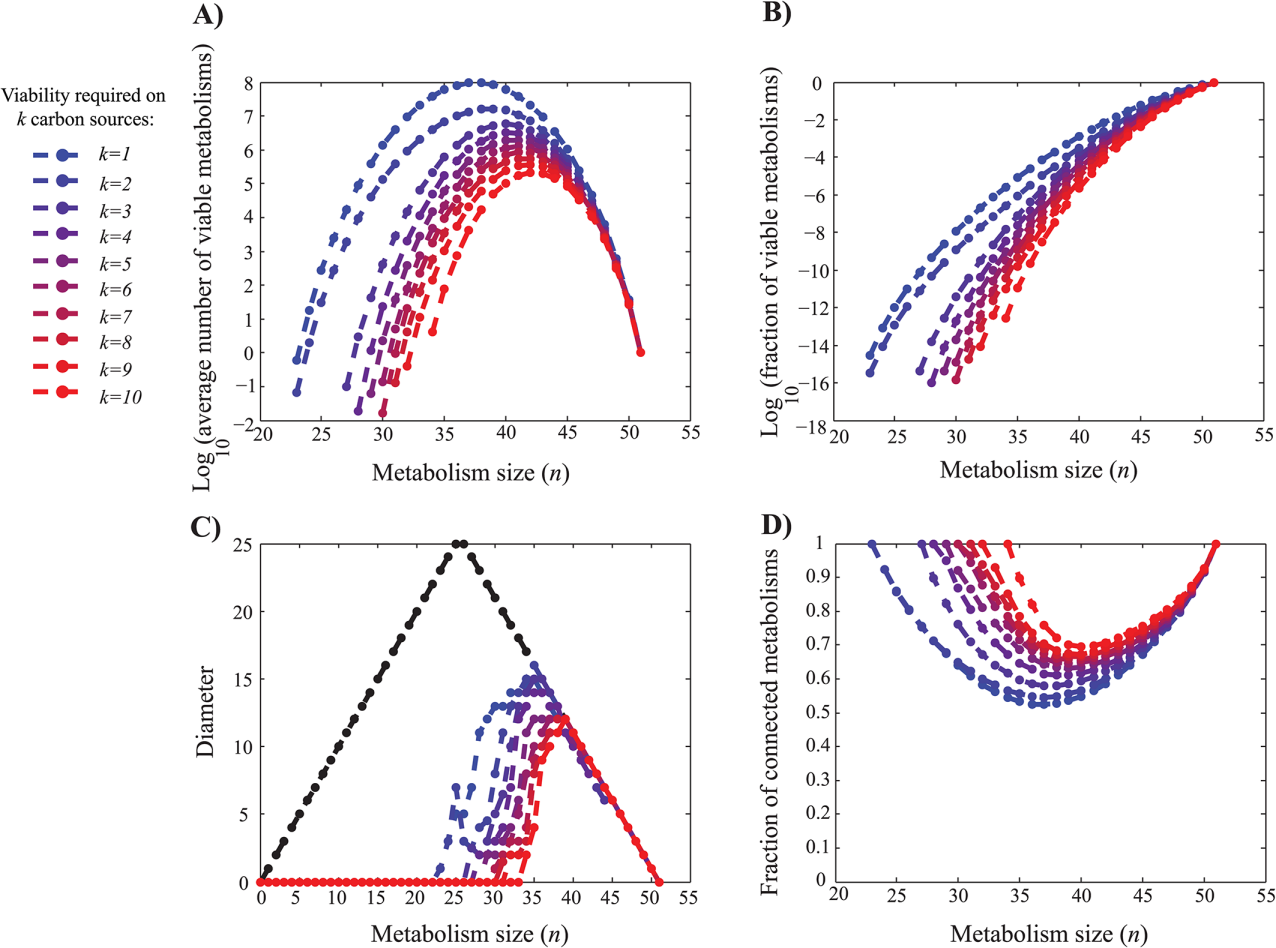
Metabolisms viable on multiple carbon sources and genotypic differences among them. **a)** Average number of metabolisms per genotype network that are viable on *k* carbon sources for 1 ≤ *k* ≤ 10. Each curve corresponds to one value of *k* and is colored as indicated in the legend. **b)** Fraction of metabolisms viable on *k* carbon sources. Note the logarithmic scale. Data on ten carbon sources in a) and b) has been previously published [19]. **c)** Genotype network diameter. Black circles indicate the diameter of genotype space for metabolisms of a given size, which is an upper bound to the diameter of any one genotype network. Colored data points indicate the median of the diameter of the genotype networks of metabolisms viable on *k* carbon sources. At *n* ≥ 40, almost all genotype networks have the maximally possible diameter. **d)** Fraction of metabolisms that are viable on *k* carbon sources and contain no disconnected reactions.

In a next analysis, we asked how the observations we made so far translate into metabolisms that are viable on some number *k* > 1 of carbon sources. This analysis is more challenging, because at each *k* there are (10k) possible *k*-tuples of carbon sources. We exhaustively enumerated, for each possible *k*-tuple of carbon sources, the number of metabolisms of a given size that are viable on that *k*-tuple. Then, we calculated the average number of metabolisms viable on a given *k*-tuple. Fig 3A and 3B show the number and fraction of viable metabolisms as a function of metabolism size *n*, and for different values of *k*. Several observations are germane. First, the unimodal relationship between the number of viable genotypes and metabolism size still holds for metabolisms viable on multiple carbon sources. Second, the smallest number of reactions in a viable metabolism increases from 23 for metabolisms viable on a single carbon source to 34 for metabolisms viable on 10 carbon sources. Third, the fraction of viable metabolisms declines at a greater than exponential rate as the number of reactions decreases (Fig 3B). Fourth, the rate of this decline becomes steeper as the number of carbon sources increases on which a metabolism is viable (Fig 3B).

S6, S7, and S8 Tables, respectively, show the median, maximum, and minimum of the number *n*_*C*_ of connected components, and the fraction of metabolisms in the largest (“giant”) component for metabolisms of size *n* required to be viable on *k* carbon sources. The results show that for metabolisms above *n* = 36 reactions, all metabolisms viable on a given number of carbon sources are connected. Wherever the set of viable metabolisms are disconnected, they are partitioned into few connected components (with a median of 2–3 and a maximum of 7), and with few exceptions most metabolisms reside in the largest of these components.

Fig 3C shows the median diameter of the set of genotypes viable on *k* carbon sources as a function of *n* (see S5A and S5B Fig, for the minimum and maximum diameters for each *k*). This median diameter is not substantially lower than for metabolisms viable on single carbon sources. Specifically, for metabolisms of most sizes, the diameter of the set of viable metabolisms lies between 5 and 15, indicating that metabolisms of the same size and viable on the same number of carbon sources can differ substantially in their reaction complement. Moreover, the median diameter as a fraction of the maximally possible diameter, i.e., the diameter of genotype space, is one or close to one for most sizes above *n*_*min*_. In other words, the set of viable metabolisms viable on any set of carbon sources is not localized to a small region of genotype space. It often spans the entire space.

Fig 3D shows not only that the majority of metabolisms considered lack disconnected reactions, but also that the fraction of metabolisms without any disconnected reactions increases with the number *k* of carbon sources on which viability is required. The patterns of organization from Fig 3A, 3B and 3C also hold for viable metabolisms where all reactions are biochemically connected to one another, as shown in S6 and S7 Figs.

### Local neighborhoods of viable metabolisms are phenotypically diverse

**Fig 4:**
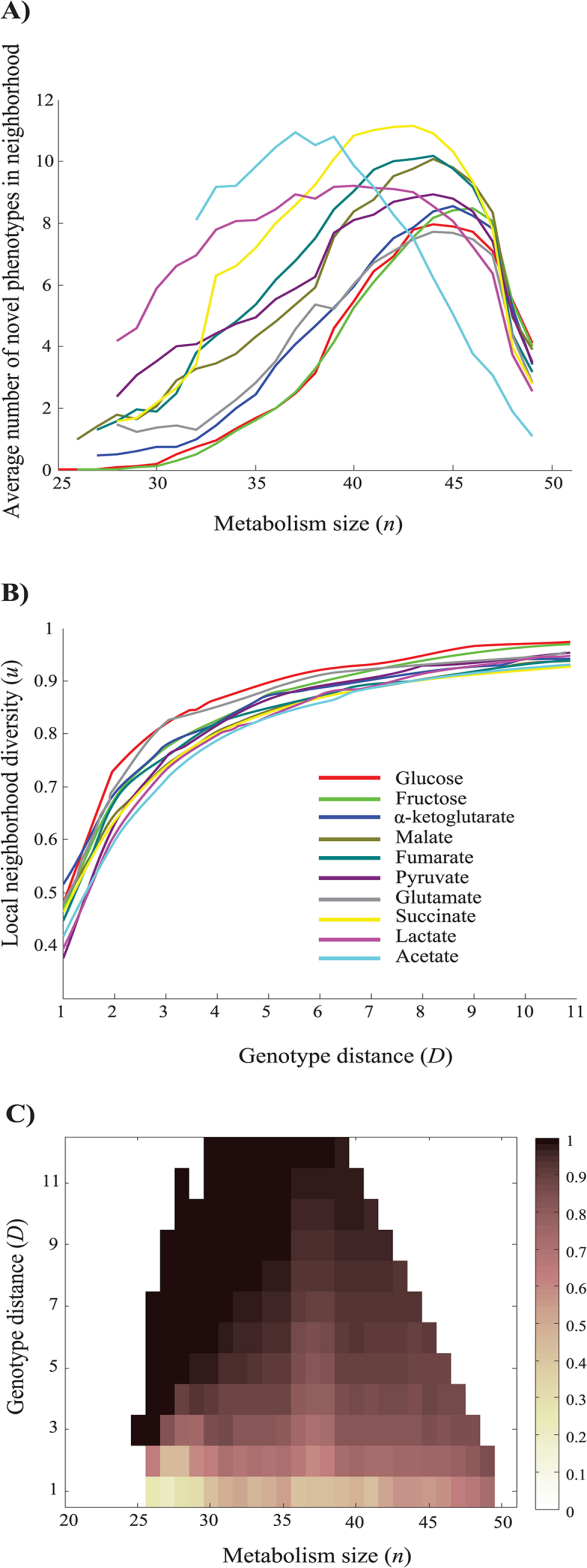
Neighborhood analysis. **a)** Average number of distinct novel metabolic phenotypes in a neighborhood as a function of metabolism size (*n*) and **b)** average local neighborhood diversity *(u)* as a function of genotypic distance (*D*) (horizontal axes), for metabolisms of size 40 that are viable on different single carbon sources, as indicated in the legend, **c)** Average local neighborhood diversity *u* (see color legend) for metabolism pairs of a given genotype distance (*D*, *y-axis*) and size (*n*, *x-axis*), that are viable on glucose, Data are based on 1000 randomly sampled networks for each metabolism size (*n*), genotypic distance (*D*), and carbon source.

We refer to the neighborhood of a metabolism *M* as the set of metabolisms differing in one reaction from *M*. Neighborhoods are important in the evolution of biological systems, because they contain those genotypes that are easily reachable through a small genotypic change–in our case, change of a single reaction- from a given genotype. In our next analysis, we studied the number of novel phenotypes contained in such neighborhoods. To this end, we first sampled 1000 metabolisms of a given size from each of the distinct genotype networks viable on different carbon sources. Representing each phenotype as a binary vector of length 10 whose *i-*th entry indicates viability (1) or inviability (0) on the *i-*th carbon source, we asked whether multiple distinct novel phenotypes in the neighborhood of a given metabolism *M* exist, i.e. phenotypes different from *M* that indicate growth on at least one additional carbon source compared to that of *M*. The answer is yes. (Fig 4A). S3 Text explains the single-peaked shape of the distributions in Fig 4A. S8 Fig shows that the number of these novel accessible phenotypes is greater than expected by chance, based on a simple randomization test.

In a next analysis, we asked whether the neighborhoods of different metabolisms viable on the same carbon source contain different novel phenotypes. If so, which novel phenotypes are accessible may depend on where in metabolic genotype space a viable metabolism is located. To find out, we randomly and uniformly sampled 1000 pairs of metabolisms with *n* reactions and viable on a given carbon source, where each pair differed in *D* reactions. Then, we determined the set of new phenotypes accessible in the local neighborhood of metabolisms *M*_1_ and *M*_2_, which we denote by *P*_1_ and *P*_2_, respectively. We then computed the fraction *u* (for *unique*) phenotypes, which appear in *P*_1_ or *P*_2_ but not in both, i.e., *u* = 1 − |*P*1 ∩ *P*2|/(|*P*1| + |*P*2| − |*P*1 ∩ *P*2). We first studied the average of *u* (regardless of *D* and *n*) for all pairs of viable metabolisms sampled, and did so for each of the 10 carbon sources. S9 Fig shows that *u* ranges from 0.65 to 0.85, indicating that the majority of novel phenotypes accessible to a local neighborhood is unique to that neighborhood, regardless of the carbon source considered. The average *u* for pairs of metabolisms where all reactions are connected to one another is very similar (S9 Fig).

Subsequently, we investigated how genotypic distance (*D*), and reaction numbers (*n*) influence phenotypic diversity *u*. We define the genotype distance as the number of reaction changes required to convert one genotype to the other. It is equal to half the Hamming distance of two genotype vectors. Fig 4B illustrates how *u* increases with increasing genotype distance *D*, for metabolisms with *n* = 40 reactions required to be viable on each of the 10 carbon sources. The figure illustrates that *u* increases rapidly until it reaches close to its maximal value at *D* ≥ 4. The dependency of *u* on *D* is qualitatively identical for different metabolism sizes (S10 Fig) and a similar trend is apparent for pairs of metabolisms viable on any one of the 10 carbon sources (S10 Fig). Fig 4C shows how *u* depends on both *D* and *n* for metabolisms viable on glucose as the sole carbon source. The figure indicates that regardless of *n*, the fraction of unique phenotypes *u* increases rapidly with increasing *D* and reaches a value close to its maximum of *u* = 1 at modest *D*. While this qualitative pattern is similar for different carbon sources and reaction numbers (S10 Fig), the quantitative relationship between *u*, *D*, and *n* depends on the carbon source (compare Fig 4C based on metabolisms viable on glucose with S11 Fig for metabolisms viable on fumarate). Despite such quantitative differences, however, a simple general pattern emerges: except for small metabolisms that are very similar to one another, the majority of phenotypes accessible from any one neighborhood are unique (*u* > 0.5). Accessible new phenotypes strongly depend on a metabolism’s location in genotype space. S12 Fig shows that for every carbon source, local neighborhood diversity *u* is greater than expected by chance based on a simple randomization test.

### Genotype networks of different phenotypes are close together in genotype space

A complementary perspective on the accessibility of novel phenotypes regards the minimal distance of the genotype networks *GN*_1_ and *GN*_2_ of different phenotypes *P*_1_ and *P*_2_, i.e., *D*_*min*_ = *min*{*D*(*G*_1_, *G*_2_)|*G*_1_ ∈ *GN*_1_ ∧ *G*_2_ ∈ *GN*_2_} where *D*(*G*_1_, *G*_2_) indicates the genotype distance of two metabolic genotypes *G*_1_ and *G*_2_. This distance is equivalent to the minimal number of reaction changes that are necessary to convert metabolisms with one phenotype into metabolisms with the other phenotype. We analyzed the distribution of this distance for genotype networks of different phenotypes, focusing our analysis on the giant component for the minority of phenotypes whose viable set of genotypes had more than one connected component (S2, S5, S6, S7 and S8 Tables). In analogy to neighboring genotypes, we call two genotype networks neighbors if their minimal distance is *D*_*min*_ = 1. Before embarking on the analysis, we note that only 84 of the 2^10^ = 1024 possible phenotypes for our 10 carbon sources have a genotype associated with it (S13 Fig). To see why, consider that all metabolisms viable on fructose are also viable on glucose, because glucose and fructose are biochemically similar and enter central carbon metabolism near one another. Therefore, there exists no metabolism viable on any combination of carbon sources that contain fructose and lack glucose (2^8^ = 256 such “forbidden” phenotypes). Analogous dependencies among other combinations of carbon sources shrink the total number of allowed phenotypes from 1024 to 84.

**Fig 5:**
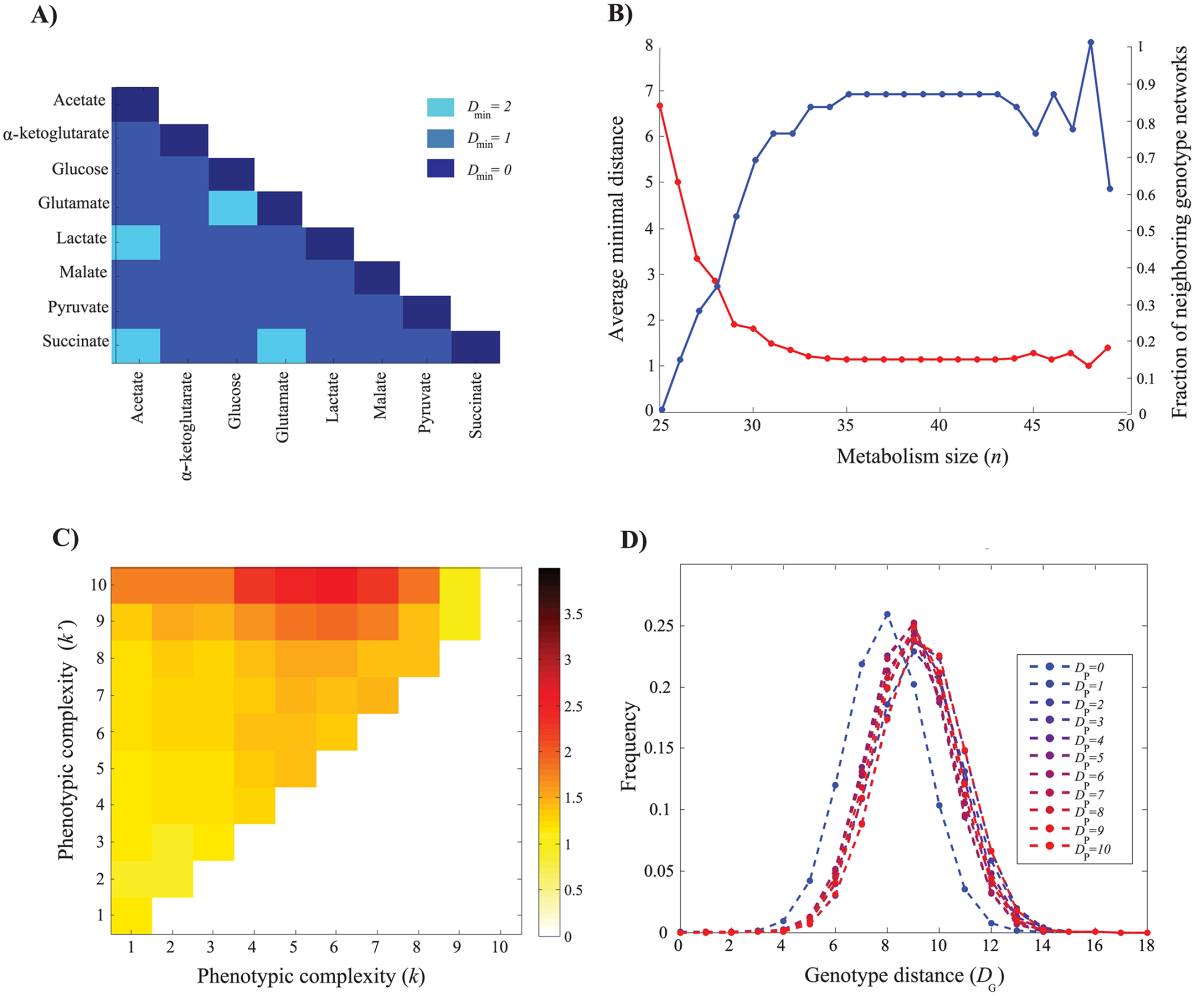
Minimal distance between genotype networks. Each rectangular colored area shows the color-coded minimal genotype distance *D*_*min*_ between genotype networks comprised of metabolisms that are viable only on carbon sources indicated on the corresponding *(x*, *y)* positions where (*n* = 35). **b)** Average minimal distance among all pairs of genotype networks (red curve, inner vertical axis) and fraction of pairs of genotype networks that are neighbors (blue curve, outer vertical axis) as a function of metabolism size (*n*). **c)** Each rectangular area shows the color-coded average of the minimal distance *D*_*min*_ (see color legend) between pairs of genotype networks with phenotypic complexity (*k*, x-axis) and (*k*’, y-axis) for metabolisms of size *n* = 35. At this size, the highest average minimal distances exists for metabolisms of complexity (*k*, *k*’) = (10, 7), which show *D*_*min*_ = 2.6. **d)** Data points of a given shading indicate the distribution of genotypic distance *D*_*G*_ (x-axis) among pairs of metabolisms with a given phenotypic distance *D*_*P*_, as indicated in the color legend for metabolisms of size *n* = 30.

We first analyzed minimal genotype distances for metabolisms viable on only a single carbon source. (Instead of ten carbon sources, there are only eight to consider in this analysis, because all metabolisms viable on fructose are also viable on glucose, and the same holds for malate and fumarate.) Fig 5A shows the minimal distance between such genotype networks at intermediate reaction numbers *n* = 35. All except 4 pairs of genotype networks have a minimal distance of one. As *n* approaches the minimally admissible size for viability, this minimal distance gets modestly larger. However, even at the lowest size (*n* = 30) where viable metabolisms exist for all carbon sources, genotype networks are immediately adjacent to one another for 19 out of the 28 possible pairs (S14 Fig). Only for a single pair of carbon sources (lactate and acetate) does the minimal distance have the largest value of *D*_*min*_ = 6 (See S14, as well as S15 Figs for two representative examples of how minimal genotype distances change with increasing metabolism size.)

Fig 5B (red curve) shows the average *D*_*min*_ between genotype networks as a function of metabolism size *n*, where the average is over all pairs of carbon sources. This average *D*_*min*_ decreases rapidly as *n* increases. Fig 5B (blue curve) shows the fraction of genotype network pairs for which *D*_*min*_ = 1 increases rapidly with increasing *n*. In sum, except for the smallest metabolisms, most genotype networks associated with viability on single carbon sources are neighbors and are thus easily reached from one another through single reaction changes.

Next, we extended this analysis to genotype networks for metabolisms viable on *k* > 1 carbon sources. For brevity, we refer to *k* as the phenotypic complexity of a metabolism. Fig 5C shows the average minimal distance, i.e., the average number of reaction changes minimally needed to reach a genotype network with phenotypic complexity *k* from a network with complexity *k*’, where *k* and *k*’ range between 1 and 10 and where *n* = 35. The figure demonstrates that *D*_*min n*_ generally increases with either *k* or *k*’, and that it is most difficult to reach metabolisms of intermediate phenotypic complexity (*k* = 7) from metabolisms with the highest complexity (*k* = 10), where *D*_*min*_ ≃ 2.6. At the same time, *D*_*min*_ decreases with increasing metabolism size (compare Figs S16A, 5C and S16B). A complementary analysis in S17 Fig, which focuses on the fraction of neighboring genotype networks (*D*_*min*_ = 1) as a function of phenotypic complexity, shows a similar pattern: At any one metabolism size, the fraction of neighboring genotype networks decreases with phenotypic complexity. S18 and S19 Figs show that these patterns also hold if we consider only those metabolisms where all reactions are connected to one another. Finally, S20 and S21 Figs show that except in the smallest metabolisms, the average of the minimal distance among all pairs of genotype networks is close to 1 (See S4 Text) indicating that most genotype networks abut each other in the genotype space.

### Genotypic similarity versus phenotypic similarity

In our last analysis, we asked how many reaction changes are required to change a metabolism with a given genotype *G*_1_ and phenotype *P*_1_ into a metabolism *G*_2_ with an arbitrary new phenotype *P*_2_. More specifically, we were interested in how the (Hamming) distance between the two genotypes *D*_*G*_ depends on the (Hamming) distance *D*_*P*_ between the phenotypes *P*_1_ and *P*_2_. *D*_*P*_ is based on the representation of phenotypes as ten-dimensional binary vectors indicating viability on each of our ten carbon sources. The answer can indicate how difficult it is to reach a distant phenotype from any one viable genotype. To compute this distance, we first sampled 1000 metabolisms with a given size (*n* = 35) among all viable metabolisms, regardless of the number of carbon sources that they are viable on. Next, for each sampled metabolism, we exhaustively calculated its genotype distance and its phenotype distance against all viable metabolisms of the same size.

The results are shown in Fig 5D. The left-most distribution shows the genotype distances of those genotypes that have identical phenotypes (*D*_*P*_ = 0), for comparison against those genotypes that have different phenotypes (*D*_*P*_>0). The figure shows that the distributions of genotypic distance of phenotypes with varying phenotypic distance are very similar. Moreover, the mean distance is shifted only slightly to the right (by one reaction change) relative to the mean distance of genotypes with identical phenotypes. Similar patterns exist for metabolisms of different sizes (S22 Fig) and for metabolisms consisting only of connected reactions (S23 Fig). Taken together, these patterns imply that reaching a genotype with an arbitrarily distant phenotype from any one point in genotype space does not require many more reaction changes than traversing a genotype network.

## Discussion

Any population of organisms evolves in a space of possible genotypes and their phenotypes. Such a “space of the possible” may harbor new and useful phenotypes, but it may also constrain a population’s potential for innovation. To understand both innovation opportunities and constraints, it is necessary to understand the organization of such spaces. For a population of organisms whose metabolic reaction network changes through addition and elimination of individual reactions, the relevant space is a space of metabolic genotypes–each genotype represents a specific set of reactions–and the phenotypes they encode. If one considers all known biochemical reactions, this space is vast, around 10^2000^ genotypes [54–56]. Past analyses thus relied on sampling [11–18]. We here complement sampling-based analyses through an exhaustive enumeration of all 2.25 x 10^15^ genotypes in the subspace whose reactions are involved in central carbon metabolism.

Our most basic observation regards the fraction of viable metabolisms, which is tiny, ranging from 10^−8^ (for acetate) to 10^−6^ (for glucose) for metabolisms viable on at least one carbon source, and becomes substantially smaller for metabolisms viable on all 10 carbon sources (10^−10^). However, because the entire space is large, these tiny fractions translate into sizable numbers of 10,850,304 and 1,549,771,520 viable metabolisms on acetate and glucose respectively and 1,029,375 metabolisms on all 10 carbon sources. Because we observe that most viable metabolisms do not contain any reactions disconnected from the rest of the reaction network, most of these variant metabolisms are not trivially obtainable by addition of disconnected reactions to a functional metabolic core. Thus, even in the modest genotype space created by all subsets of 51 biochemical reactions, there are myriad alternative metabolic organizations that achieve viability through different means.

The fraction of viable metabolisms is not uniformly distributed among metabolisms with different complexity, i.e., number *n* of reactions. Regardless of the specific phenotype one considers, the fraction has a minimum at the minimal complexity needed for viability (the smallest *n*_*min*_ = 23 exists for viability on glucose, and the largest *n*_*min*_ = 30 for metabolisms viable on acetate) and increases rapidly towards a fraction of one for the largest metabolisms. In addition, metabolisms with the same phenotype form large connected networks. These networks may contain more than one connected components at the smallest complexity but they congeal to a single component at intermediate complexity. Moreover, this “giant” component [57,58] extends increasingly far through genotype space until its diameter becomes equal to the entire space at intermediate reaction numbers. Taken together, this means that central carbon metabolism shows substantial internal flexibility in its organization. It can be altered one reaction at a time to create different metabolic architectures with the same phenotype, exemplified by metabolisms like those shown in Fig 2. that differ in about half of their reactions. The exceptions to this rule can be found among the smallest, least complex metabolisms. Evolutionary change that alters metabolic genotypes without altering phenotypes is easier in complex metabolisms with many reactions.

A metabolism’s complexity is also relevant for its potential to encounter novel metabolic phenotypes in its immediate neighborhood, i.e., through single reaction changes. The number of different novel phenotypes that are encountered in a metabolism’s neighborhood exceeds one for all but the smallest metabolisms, and it rises to a maximum of 8–11 at intermediate metabolic complexity. That it does not increase further for larger *n* is the result of a model limitation, namely that we consider only ten carbon sources. Large metabolisms are already viable on most of these carbon sources, such that further addition of reactions can no longer create novel phenotypes that are viable on additional carbon sources (S24 Fig). These observations suggest that the number of novel phenotypes accessible through single reaction changes increases with metabolic complexity.

While the neighborhoods of most metabolisms contain multiple novel phenotypes, the identity of these phenotypes depends strongly on a metabolism’s location in genotype space. That is, the majority of novel phenotypes contained in the neighborhoods of two closely related metabolisms are not shared between these neighborhoods. In other words, any one novel phenotype tends to occur either in one or the other neighborhoods, but not in both. This means that the evolutionary potential of any one metabolism with a given phenotype is contingent upon its genotype. This contingency is alleviated by the connectedness of different metabolisms with the same phenotype. Because their genotype can be altered without phenotypic change, phenotype-preserving evolutionary change in genotypes can make different neighborhoods and their novel phenotypes accessible. In this regard, it is also relevant that the minimal distance of most genotype networks is small, such that one can reach novel phenotypes through one or few genotypic changes from any one genotype network. The exceptions to this rule come again from the smallest metabolisms, suggesting that metabolic complexity also facilitates this aspect of phenotypic evolution.

One major obstacle to genotype-phenotype mapping comes from the vast size of genotype spaces. We could overcome this obstacle here by considering a modestly sized genotype space of 10^15^ carbon metabolism variants. Carbon metabolism is an attractive small study system, because it plays a key role in extracting energy from extracellular carbon sources. In addition, we limited ourselves to viability on 10 different carbon sources, and thus to potentially 2^10^ = 1024 viability phenotypes. This number is sufficiently small to be computationally tractable, yet large enough to allow quantitative analyses, for example about the phenotypic diversity of different neighborhoods. Even so, we could enumerate the phenotype of all 10^15^ metabolisms only after taking advantage of certain relationships among metabolisms, such as that the children of inviable metabolisms are also inviable.

A limitation of analyzing central carbon metabolism is that it is not suited to study metabolic innovation in essential nutrients like nitrogen or sulfur. It thus remains to be seen whether similar principles hold for these nutrients and their metabolism. Sampling studies of larger genotype spaces suggest that this is indeed the case [14,59].

Another limitation of our analysis is its focus on evolutionary constraints that are imposed only by the presence or absence of reactions. In other words, we have neglected regulatory constraints that can arise through suboptimal expression or regulation of an enzyme. In this regard, we note that such constraints are most important if one focuses on the quantitative predictions of biomass growth via flux balance analysis [60]. In contrast, we here focus on the purely qualitative prediction of viability, i.e., whether biomass can be produced at all. This qualitative phenotype is biologically relevant if one considers that many organisms grow slowly in their native environment [61–63]. In addition, we note that regulatory constraints can easily be broken in evolution, even on the short time scales of laboratory evolution experiments [60,64,65].

A third limitation comes from the fact that the exhaustive enumeration we pursue requires us to start from a limited “universe” of chemical reactions. The choice of reactions in this universe may introduce some biases into our analysis. For example, it is not clear whether the small number of metabolisms viable on acetate is a result of this choice, or whether it would also persist in an unbiased analysis of larger, genome scale metabolisms. However, we note that our core results agree well with previous studies based on sampling of genome-scale metabolisms that comprise more than a thousand reactions. For instance, genome scale metabolisms with the same phenotype can be genotypically very different [12] and usually form single connected genotype networks [19], which is in line with our present observation that genotype networks have large diameter. Moreover, genome-scale metabolisms can encounter many new phenotypes in their immediate neighborhood, and the neighborhood of different genome-scale metabolisms contains different novel phenotypes [12,14,59], which is consistent with our neighborhood analysis. Finally, genome-scale metabolisms with different phenotypes can be found close together in genotype space [12,59].

A final limitation comes from our definition of a metabolic genotype centered on individual metabolic reactions. Although widely used in the field [44, 66–70] this notion of a genotype does not take into account that some reactions are catalyzed by multiple enzymes [71], and conversely, that some enzymes catalyze multiple reactions [72–74]. Our focus on the metabolic reaction as the most elementary unit of evolutionary change should not distract from the fact that actual change in metabolic systems may be more complex.

In sum, our exhaustive analysis of central carbon metabolism’s genotype space reveals an organization that is conducive to both the preservation of phenotypes in the face of genotypic change, and the exploration of new phenotypes. Because metabolisms with the same phenotype can be connected to one another through single reaction changes, viability phenotypes can be preserved through substantial genotypic change. Because connected sets of genotypes associated with different phenotypes are close to each other in genotype space, novel phenotypes can often be reached with few or no transitions through intermediate phenotypes. These principles only break down in metabolisms with low complexity, close to the minimal number of reactions needed for viability. Thus, increasing metabolic complexity enhances both the potential for evolutionary conservation and innovation.

## Methods

### Flux balance analysis

Flux balance analysis (FBA) is a constraint-based computational modeling approach that is widely used for quantitative analysis and modeling of metabolism. FBA predicts the metabolic flux through every reaction in a metabolism, based on information about the metabolism’s reactions, as well as about the stoichiometric coefficients of the reactants in each reaction. These coefficients are contained in the stoichiometric matrix *S*, which is of dimension *m*×*n*, where *m* and *n*, respectively, denote the number of metabolites and the number of reactions in the metabolism. An important assumption behind FBA is that the concentration of metabolites does not change, that is, the metabolism is in a steady state. This assumption imposes mass conservation constraints on the metabolites in the network, which can be mathematically expressed as *Sv* = 0, where *v* is the vector of metabolic fluxes *v*_*i*_ through reaction *i*. The possible solutions of the above equation are the allowable flux vectors, which form the null space of the stoichiometric matrix *S*. This space is further constrained by the fact that each reaction has a maximally and minimally possible flux through it. FBA uses an optimization technique called linear programming to identify among the allowed flux vectors those vectors that maximize an objective function *Z*. This task can be formulated as finding a flux vector v* with the property

$$v^{*}=max_{v}\;Z\left(v\right)=\{c^{T}v|S.v=0,\;a\leq v\leq \;b\}$$

where the vector *c* is a set of scalar coefficients representing the maximization criterion, and each entry *a*_*i*_ and *b*_*i*_ of vectors *a* and *b*, respectively, indicates the minimally and maximally possible flux through reaction *i*.

We are interested in knowing whether a metabolic reaction network can sustain life in a given environment, that is, whether it can synthesize all essential small biomass molecules required for survival and growth. In this work, we used 13 well-known precursor substances from central carbon metabolism as the set of required biomass molecules (S9 Table). As is common in FBA applications [75–79], the objective function that we use for the linear programming of FBA is a biomass reaction that transforms 13 precursors into biomass (S9 Table). We used the package CLP (1.4, Coin-OR; https://projects.coin-or.org/Clp) to solve linear programming problems.

### Chemical environments

In addition to a stoichiometric matrix and an objective function (biomass growth), it is necessary to define the chemical composition of an environment that contains different nutrients required for the synthesis of biomass precursors. We consider minimal growth environments composed of a sole carbon source, along with oxygen, ammonium, inorganic phosphate, sulfate, sodium, potassium, cobalt, iron (Fe^2+^ and Fe^3+^), protons, water, molybdate, copper, calcium, chloride, magnesium, manganese and zinc. All these nutrients except the carbon source are shared between different minimal environments. Each minimal environment contains a different one of the 10 carbon sources acetate, *α*-ketoglutarate, fumarate, fructose, glucose, glutamate, lactate, malate, pyruvate, and succinate in our analysis.

### Reaction universe

The “universe” of reactions in our metabolic genotype space is based on *E*. *coli* central carbon metabolism [80], from which we deleted four reactions involved in ethanol synthesis, metabolism, and transport. We also grouped the reactions catalyzed by aconitase A and aconitase B into one reaction, to render exploration of all metabolisms that consist of different combinations of these reactions feasible. The final reaction set consists of *N* = 51 intracellular reactions that can be present or absent in different metabolisms (S9 Table). Twenty different transport reactions, which are necessary to import nutrients or excrete waste products, are present in all metabolisms we study, i.e., we do not vary their presence among metabolisms.

### Metabolic genotypes, metabolic phenotypes and genotype networks

The nucleotide sequence of the genes encoding the enzymes catalyzing a metabolism’s reactions constitutes the metabolic genotype of an organism. However, for our purpose, we use a more compact representation of a metabolic genotype, in which we represent this genotype as a binary vector whose *i*-th entry corresponds to the *i*-th reaction in our reaction universe. The *i*-th entry is equal to one if an organism’s genome encodes an enzyme capable of catalyzing this reaction, and zero otherwise. The genotype space of all possible metabolisms comprises *2* ^*N*^ metabolisms (*N* = 51). Each metabolism can be thought of as a point in this space. We call metabolisms that are able to synthesize all 13 biomass precursors from nutrients in this environment *viable*. More precisely, we consider a metabolism viable on a carbon source if its biomass synthesis rate is greater than one percent of the biomass synthesis rate of the network formed by all *N* = 51 reactions on that carbon source [19]. Our definition of a carbon utilization phenotype for any one genotype is based on its viability on different carbon sources. Specifically, we assign to each genotype a phenotype vector of length 10, equal to the number of distinct carbon sources we consider. The *i-*th entry of the phenotype vector corresponds to the minimal environment containing the *i*-th sole carbon source. This entry equals 1 if the metabolism is viable on that minimal environment and zero otherwise. In other words, we consider 2^10^ = 1024 distinct metabolic phenotypes. We partition metabolic genotype space into distinct sets of genotypes, each with a different phenotype. Each such genotype set can be further partitioned into subsets of metabolisms with different sizes, that is, different numbers *n* of chemical reactions. If a subset of metabolisms (genotypes) forms a connected graph [57,58] in genotype space, we call that graph a genotype network. For some analyses, it is useful to consider a modified definition of a phenotype that just specifies whether a metabolism is viable on *at least* a specific set of carbon sources–it may be viable on other carbon sources as well. With this phenotype definition, different genotype sets and genotype networks can overlap.

### Exhaustive enumeration of viable metabolisms

To exhaustively characterize the phenotype of every single one among 2^51^ (10^15^) metabolic genotypes one would need to use FBA 10^15^ times. Given that a typical FBA computation takes of the order of 10^−2^ seconds of CPU time, exhaustive computational phenotyping would require 10^5^ years and would thus be prohibitive. However, one can take advantage of two simple facts to render this computation feasible in approximately 10 days [19,81]. First, six among the 51 internal reactions of central carbon metabolism are essential for viability on every carbon source we consider [15]. The corresponding entries of the genotype vector need to be set to one, which reduces the number of required FBA computations by a factor 2^6^ from 2^51^ (10^15^) to 2^45^ (10^13^).

Second, all metabolisms (“children”) that contain a subset of the reactions of an inviable metabolism (“parent”) will also be inviable, because deleting one or more reactions from an inviable metabolism cannot result in a viable metabolism. In an earlier work, one of us designed an algorithm to take advantage of this observation [81]. It divides genotype vectors of length 45 into 5 distinct sub-vectors of length 9, determines all viable genotypes originating from each binary sub-vector of length 9 (2^9^ = 512 subvectors), and merges the sub-vectors of viable genotypes in a five-step procedure to determine all the viable genotypes on a given carbon source. At each step, only sub-vectors that preserve viability are merged, which dramatically decreases the number of required FBA tests to approximately10^9^ total tests.

Once the set *GN*(*C*_*i*_) of metabolisms viable on each of the 10 carbon sources *C*_*i*_ has been determined, one can easily identify the set of metabolisms that are viable on a given subset *S* of the 10 carbon sources. Specifically, *V*(*S*) = {*G* ∈ Ω, ∀ *C*_*i*_ ∈ *S*|*G* ∈ *GN*(*C*_*i*_)} where *G* denotes a given genotype belonging to the genotype space Ω. Similarly, one can define the set of genotypes that are exclusively viable on carbon sources in *S* as: *V*(*S*) = {*G* ∈ Ω, ∀ *C*_*i*_ ∈ *S*, ∀ *C*_*j*_ ∈ *S*′|*G* ∈ *GN*(*C*_*i*_), *G* ∉ *GN*(*C*_*j*_)}, where *S’* denotes the complement of *S*.

### Connectedness of metabolic genotype networks

We can represent each set of metabolisms (genotypes) of a given size *n* that is viable on a subset *S* of carbon sources as a graph. The nodes of this graph are metabolisms. Two viable metabolisms *A* and *B* are connected by an edge if metabolism *A* is convertible to *B* via a reaction swap, that is, by deleting a reaction that *A* possesses but *B* lacks, followed by adding a reaction that *B* possesses but *A* lacks. We note that such a swap leaves the number of reactions constant, as is required for metabolisms that have the same size. However, any one reaction swap can be decomposed into an addition of a reaction followed by a deletion of a reaction. In other words, viable metabolisms that are neighbors based on reaction swaps are also connected through single reaction changes.

If this graph of metabolisms is connected, then every single metabolism in it can be reached from any other metabolism via a sequence of single reaction changes. Otherwise, the graph fragments into several disconnected components, one of which may be much larger than the others, and is therefore often also called the “giant” component [57,58]. The connected components of a graph can be computed with the aid of algorithms like Breadth-First Search (BFS) [82]. BFS requires a graph’s adjacency list, which contains the neighbors of each node in the graph. To generate this list for a genotype network, one needs to compare *V* ^2^ pair of metabolisms to ascertain whether they are neighbors. Because the genotype networks we consider may comprise many thousands to millions of metabolisms, doing so would be computationally prohibitive. Moreover, storing an entire adjacency list causes memory problems in large genotype networks. Therefore, we developed an algorithm to examine connectedness of genotype networks [81], which differs from conventional BFS in that (i) it does not need to fill the adjacency matrix in advance, and (ii) it can avoid comparing genotypes that could not possibly be neighbors. In doing so, it reduces the number of genotype comparisons from *V* ^2^ to *mV* where *m* is the average number of a genotype’s neighbors. Using this algorithm, we could determine connectedness of genotype networks comprising as many as 10^6^ metabolisms. For larger metabolisms, where the requirements for storing all metabolisms becomes prohibitive, we could determine genotype network connectivity by taking advantage of the following simple principle: If a genotype network of metabolisms of size *n* is connected, then the genotype network of metabolisms of size *n* + 1, each genotype of which is constructed by adding an additional reaction to each genotype belonging to the genotype networks of size *n*, is also connected [19]. In other words, we could infer the connectedness of larger genotype networks from the connectedness of smaller ones.

### Diameter of metabolic genotype networks

The diameter of a graph (genotype network) is the maximum length of all shortest paths between any pair of nodes that reside in the same connected component. In a connected genotype network, the shortest path between any pair of genotypes is the minimal number of reaction changes required to convert the two genotypes into each other. This number is equivalent to half of the Hamming distance between the binary vectors representing the genotypes. To determine the diameter of a genotype network, we needed to identify those genotypes whose Hamming distance is maximal. We were able to do this through exhaustive enumeration for genotype networks with fewer than 10^5^ metabolisms. For larger genotype networks, we could only determine a lower bound on the diameter, and we did so by sampling 10^5^ genotypes from a given genotype network and determining the two genotypes with the largest distance among them. We note that maximum diameter of genotype space as a function of metabolism size (*n*) is *Min*{(51 − *n*), *n*}, and in most of the large genotype networks, the sampling based diameter estimate was equal to the maximum diameter of the genotype space. This confirms that the sample size was big enough to accurately estimate the diameter of the genotype networks

## Supporting Information

S1 Text Size differences between minimal metabolisms viable on acetate and glucose. (DOCX)

S2 Text Prediction of the number of viable metabolisms based on binomial coefficients. (DOCX)

S3 Text The unimodal distribution of the number of novel phenotypes in the neighborhood of viable metabolisms. (DOCX)

S4 Text Minimal genotype network distance as a function of phenotypic complexity and metabolism size. (DOCX)

S1 Fig Central carbon metabolism. (EPS)

S2 Fig Example minimal metabolisms. (EPS)

S3 Fig Binomial distribution of the number of viable metabolisms. (EPS)

S4 Fig Viability of metabolisms that contain no disconnected reactions on different carbon sources and the genotypic differences among them. (EPS)

S5 Fig Genotype network diameter for metabolisms viable on multiple carbon sources. (EPS)

S6 Fig Viability of metabolisms that contain no disconnected reactions on multiple carbon sources. (EPS)

S7 Fig Diameter of genotype networks of metabolisms that contain no disconnected reactions and that are viable on multiple carbon sources. (EPS)

S8 Fig Comparison of the number of novel phenotypes in a neighborhood between actual genotype networks and randomized (null) networks. (EPS)

S9 Fig Average local neighborhood diversity. (EPS)

S10 Fig Local neighbor diversity as a function of genotypic distance. (EPS)

S11 Fig Average local neighborhood diversity. (EPS)

S12 Fig Comparison of the local neighborhood diversity (*u*) between actual genotype networks and randomized (null) networks. (EPS)

S13 Fig Number of phenotypes as a function of metabolism size (*n*). (EPS)

S14 Fig Minimal distance between genotype networks. (EPS)

S15 Fig Minimal distance between genotype networks as a function of metabolism size. (EPS)

S16 Fig Minimal distance between genotype networks as a function of phenotypic complexity. (EPS)

S17 Fig Fraction of neighboring genotype networks as a function of phenotypic complexity. (EPS)

S18 Fig Minimal distance between genotype networks as a function of phenotypic complexity for metabolisms without disconnected reactions. (EPS)

S19 Fig Fraction of neighboring genotype networks as a function of phenotypic complexity. (EPS)

S20 Fig Average minimal distance and fraction of neighboring genotype networks as a function of metabolism size. (EPS)

S21 Fig Average minimal distance and fraction of neighboring genotype networks as a function of metabolism size. (EPS)

S22 Fig Distribution of genotypic distances *D*_*G*_ for phenotypes of a given distance *D*_*P*_. (EPS)

S23 Fig Distribution of genotypic distances *D*_*G*_ for phenotypes of a given distance *D*_*P*_ (only connected metabolisms). (EPS)

S24 Fig Normalized number of novel phenotypes in neighborhood. (EPS)

S1 Table Further information on the number of viable metabolisms and minimal metabolisms. (DOCX)

S2 Table Number of connected components and the fractional size of the largest component for the metabolisms viable on a given carbon source. (DOCX)

S3 Table Percentage of metabolism pairs with genotypic distance equal to network diameter, where different members of a metabolism pair do not use alternative reactions that differ only in a co-factor. (DOCX)

S4 Table Minimum number of blocked reactions among all pairs of metabolisms with genotypic distance equal to genotype network diameter. (DOCX)

S5 Table Number of connected components and the fractional size of the largest component for the metabolisms viable on a given carbon source. The information in this table is restricted to metabolisms without disconnected reactions.
(DOCX)

S6 Table Median of the number of connected components and the fractional size of the largest component for metabolisms viable on multiple carbon sources. (DOCX)

S7 Table Maximum number of connected components and the fractional size of the largest component for metabolisms viable on multiple carbon sources. (DOCX)

S8 Table Minimum number of connected components and the fractional size of the largest component for metabolisms viable on multiple carbon sources. (DOCX)

S9 Table Metabolites and reactions in central carbon metabolism. (XLSX)
