# Supplementary material for: Exhaustive Analysis of a Genotype Space Comprising 1015 Central Carbon Metabolisms Reveals an Organization Conducive to Metabolic Innovation
Source: PLoS Comput Biol. 2015 Aug 7;11(8):e1004329. doi: 10.1371/journal.pcbi.1004329 (PMC4529314; doi:10.1371/journal.pcbi.1004329)
Supplement: S1 Text — (DOCX) [file pcbi.1004329.s001.docx]

**S1 Text: Size differences between minimal metabolisms viable on acetate and glucose**

As we mentioned in the main text, the minimal number of reactions in a viable metabolism (i.e. minimal metabolism) is not the same for different carbon sources. For example, it varies from *n_min_=23* for glucose and fructose to *n_min_=30* for acetate (S2 Fig.).The reason why a minimal metabolism using acetate needs more reactions is that it uses reactions from gluconeogenesis (S2b Fig.) whereas glucose metabolism relies on glycolysis (S2A Fig.). More specifically, the acetate (ac) to be metabolized is converted to acetyl-coenzyme A (accoa, S2B Fig.) through two consecutive reactions, catalyzed respectively by acetate kinase and phosphotransacetylase. Acetyl-coenzyme A is then used in the glyoxylate cycle in order to produce malate and oxaloacetate. The latter is subsequently converted to phosphoenolpyruvate by phosphoenolpyruvate carboxylase. The gluconeogenic pathway that ensures production of the remaining essential biomass molecules is initiated from phosphoenolpyruvate. Thus, the two reactions in the production of acetyl-coenzyme A, plus the five reactions needed to produce phosphoenolpyruvate through the glyoxylate cycle (i.e. isocitrate lyase, malate synthase, succinate dehydrogenase, fumarase, and malate dehydrogenase) account for the seven additional reactions that are required for viability of a minimal metabolism on acetate. In contrast, glucose neither needs the reactions of the glyoxylate shunt nor those of acetate metabolism, and can thus be metabolized with fewer reactions.
