# Supplementary material for: Exhaustive Analysis of a Genotype Space Comprising 1015 Central Carbon Metabolisms Reveals an Organization Conducive to Metabolic Innovation
Source: PLoS Comput Biol. 2015 Aug 7;11(8):e1004329. doi: 10.1371/journal.pcbi.1004329 (PMC4529314; doi:10.1371/journal.pcbi.1004329)
Supplement: S2 Text — (DOCX) [file pcbi.1004329.s002.docx]

**S2 Text: Prediction of the number of viable metabolisms based on binomial coefficients**

S3A and S3B Figs illustrate that a shifted binomial coefficient (i.e.,) qualitatively predicts the relationship between reaction number and the number of metabolisms viable on a carbon source. However, it overestimates this number, especially for metabolisms at low- and intermediate sizes. The reason for the qualitative agreement stems from the fact that adding reactions to a viable metabolism will not render this metabolism inviable. Assume that only a single viable metabolism with a set of $n_{\min}$reactions exists for a given carbon source. Adding any subset of the remaining $N-n_{\min}$reactions to this metabolism will not render it inviable. To obtain a viable metabolism with size *n* that lies $n-n_{\min}$reactions above the minimum, one has possible choices of $n-n_{\min}$ reactions, which explains the qualitative binomial relationship.

The discrepancies between the binomial relationship and the data stem from violations of this assumption. In previous contributions [1,2] we showed that there are usually multiple minimal viable metabolisms$v_{\min}$. For instance, there are 3 and 4 minimal viable metabolisms on glucose and acetate, respectively (Table S1). If one extends the above line of reasoning to incorporate this observation, one arrives at the relationship $v_{\min}\left(_{n-n_{\min}}^{N-n_{\min}}\right)$ as a predictor for the number of viable metabolisms, which is shown in dashed lines in S3A an S3B Figs. This predictor is clearly superior to the shifted binomial coefficient, but a slight discrepancy persists.

This discrepancy has two causes, one a source of underestimating, the other a source of overestimating numbers of viable metabolisms. To explain them, we briefly review some previous observations on minimal metabolisms [1,3,4]. By definition, a minimal metabolism is one from which no reaction can be removed without eliminating viability. Importantly, a minimal metabolism is not necessarily the smallest possible viable metabolism, because there may be metabolisms with more than $n_{\min}$reactions, from which no reactions can be removed. On glucose, for example, the smallest viable metabolisms with $n_{\min}=23$reactions is also a minimal metabolism, but there also exist other metabolisms, at sizes *n=24* (8 metabolisms), at *n=25* (23 metabolisms), up through *n=30*, from which no reaction can be removed. To each of these metabolisms, any number of reactions can be added without abolishing viability, and each of them can thus contribute to the number of viable metabolisms at larger sizes. Not taking them into account is a reason why the predictor $v_{\min}\left(_{n-n_{\min}}^{N-n_{\min}}\right)$ underestimates the total number of viable metabolisms at these sizes.

To understand how the binomial predictor can also overestimate the number of viable metabolisms, consider two minimal metabolisms A and B of the same size, and the metabolism AB consisting of the union of their constituent reactions. Because AB can be viewed as resulting from adding sets of reactions to A or to B, it should be viable ($AB=A\cup\left(AB\backslash A\right)$, $AB=B\cup\left(AB\backslash B\right)$). The above binomial expression$v_{\min}\left(_{n-n_{\min}}^{N-n_{\min}}\right)$ implicitly counts the metabolism AB twice in computing the number of viable metabolisms, whereas AB should only be counted once. This cause is responsible for the overestimation of total metabolism sizes at large *n*, for example at *n=49* to *n=51* for glucose.

**References:**

1. Barve A, Hosseini S-R, Martin OC, Wagner A (2014) Historical contingency and the gradual evolution of metabolic properties in central carbon and genome-scale metabolisms. BMC Syst Biol 8: 48.

2. Hosseini S-R (2013) Exhaustive genotype-phenotype mapping in metabolic genotype

space. M.Sc. Thesis, Swiss Federal Institue of Technology Zürich. Available:

<http://e-collection.library.ethz.ch/view/eth:7522?q=(keywords_en:PHENOTYPE>.

Accessed 14 November 2013.

3. Matias Rodrigues JF, Wagner A (2011) Genotype networks, innovation, and robustness in sulfur metabolism. BMC Syst Biol 5: 39.

4. Bilgin T, Wagner A (2012) Design constraints on a synthetic metabolism. PLoS One 7: e39903.
