# Supplementary material for: Exhaustive Analysis of a Genotype Space Comprising 1015 Central Carbon Metabolisms Reveals an Organization Conducive to Metabolic Innovation
Source: PLoS Comput Biol. 2015 Aug 7;11(8):e1004329. doi: 10.1371/journal.pcbi.1004329 (PMC4529314; doi:10.1371/journal.pcbi.1004329)
Supplement: S3 Text — (DOCX) [file pcbi.1004329.s003.docx]

**S3 Text: The unimodal distribution of the number of novel phenotypes in the neighborhood of viable metabolisms**

To explain the unimodal distribution from 4A Fig, consider first metabolisms *M* whose size *n* is below this peak. As we mentioned in the text, the fraction of viable metabolisms increases faster than exponentially with increasing reaction numbers (1B Fig.). At larger sizes, more metabolisms in any one metabolism’s neighborhood are thus viable, which also increases the number of novel phenotypes that these metabolisms can have. This observation can explain that the number of accessible novel phenotypes increases with *n*, at least up to intermediate *n*. The fraction of all viable metabolisms (1B Fig.) continues to increase above the value of *n* where the number of accessible novel phenotypes is maximal, but a second pattern becomes important above this peak *n*. Specifically, the total number of distinct phenotypes that all metabolisms of a given size can have is highest for intermediate sizes and decreases at the largest sizes. The reason is that metabolisms *M* containing most reactions also tend to be viable on most carbon sources, such that there are fewer possible phenotype vectors with more ones than *M* (S11 Fig.). This is why the average number of accessible novel phenotypes declines beyond the peak *n*.
