# Supplementary material for: Exhaustive Analysis of a Genotype Space Comprising 1015 Central Carbon Metabolisms Reveals an Organization Conducive to Metabolic Innovation
Source: PLoS Comput Biol. 2015 Aug 7;11(8):e1004329. doi: 10.1371/journal.pcbi.1004329 (PMC4529314; doi:10.1371/journal.pcbi.1004329)
Supplement: S4 Text — (DOCX) [file pcbi.1004329.s004.docx]

**S4 Text: Minimal genotype network distance as a function of phenotypic complexity and metabolism size**

We determined the average minimal distance (*D_min_*) between the genotype networks of phenotypes with a given complexity *(k=k’),* as a function of metabolism size *n*. Except for the smallest metabolisms (*n<35*), the average minimal distance is close to *D_min_*=1, regardless of phenotypic complexity. Similarly, the fraction of neighboring genotype networks is only low in small metabolisms *(n<35)*, and this fraction increases until it reaches a maximum at intermediate metabolism sizes (S18 Fig.). At these sizes, the fraction of neighboring genotype networks depends on the phenotypic complexity, decreasing from lowest to highest complexity. Genotype networks of phenotypes with lower phenotypic complexity tend to be closest. S19 Fig indicates that the same patterns obtain when we consider only those metabolisms where all reactions are connected to one another.
