# Supplementary material for: Exhaustive Analysis of a Genotype Space Comprising 1015 Central Carbon Metabolisms Reveals an Organization Conducive to Metabolic Innovation
Source: PLoS Comput Biol. 2015 Aug 7;11(8):e1004329. doi: 10.1371/journal.pcbi.1004329 (PMC4529314; doi:10.1371/journal.pcbi.1004329)
Supplement: S1 Table — (DOCX) [file pcbi.1004329.s029.docx]

| Carbon source | *n_min_* | number of viable metabolisms at *n_min_* | total number of viable metabolisms | *n_max_* | maximum number of viable metabolisms at *n_max_* |
| --- | --- | --- | --- | --- | --- |
| Acetate | 30 | 4 | 10850304 | 40 | 1922772 |
| α-ketoglutarate | 25 | 6 | 928636928 | 38 | 153139863 |
| Fructose | 23 | 3 | 1473094400 | 37 | 227234491 |
| Fumarate | 26 | 8 | 412139520 | 38 | 68804412 |
| Glucose | 23 | 3 | 1549771520 | 37 | 239328665 |
| Glutamate | 26 | 8 | 342265856 | 38 | 56385611 |
| Lactate | 26 | 2 | 141944832 | 39 | 23190620 |
| Malate | 25 | 8 | 781627392 | 38 | 126729791 |
| Pyruvate | 26 | 6 | 353501184 | 38 | 57805322 |
| Succinate | 27 | 8 | 217774080 | 39 | 36626028 |
