# Supplementary material for: Exhaustive Analysis of a Genotype Space Comprising 1015 Central Carbon Metabolisms Reveals an Organization Conducive to Metabolic Innovation
Source: PLoS Comput Biol. 2015 Aug 7;11(8):e1004329. doi: 10.1371/journal.pcbi.1004329 (PMC4529314; doi:10.1371/journal.pcbi.1004329)
Supplement: S2 Table — (DOCX) [file pcbi.1004329.s030.docx]

| *n* | Acetate | | Alpha-ketoglutarate | | Fructose | | Fumarate | | Glucose | | Glutamate | | Lactate | | Malate | | Pyruvate | | Succinate | |
| --- | --- | --- | --- | --- | --- | --- | --- | --- | --- | --- | --- | --- | --- | --- | --- | --- | --- | --- | --- | --- |
|  | *n_C_* | *r_G_* | *n_C_* | *r_G_* | *n_C_* | *r_G_* | *n_C_* | *r_G_* | *n_C_* | *r_G_* | *n_C_* | *r_G_* | *n_C_* | *r_G_* | *n_C_* | *r_G_* | *n_C_* | *r_G_* | *n_C_* | *r_G_* |
| 23 |  |  |  |  | 2 | 0.6666 |  |  | 2 | 0.6666 |  |  |  |  |  |  |  |  |  |  |
| 24 |  |  |  |  | 2 | 0.6373 |  |  | 2 | 0.6373 |  |  |  |  |  |  |  |  |  |  |
| 25 |  |  | 1 | 1 | 2 | 0.9969 |  |  | 2 | 0.9969 |  |  |  |  | 1 | 1 |  |  |  |  |
| 26 |  |  | 2 | 0.9672 | 2 | 0.9919 | 1 | 1 | 2 | 0.9920 | 1 | 1 | 2 | 1 | 2 | 0.9642 | 3 | 0.3333 |  |  |
| 27 |  |  | 2 | 0.9973 | 1 | 1 | 2 | 0.9629 | 1 | 1 | 3 | 0.9425 | 1 | 0.9622 | 1 | 1 | 4 | 0.9617 | 1 | 1 |
| 28 |  |  | 3 | 0.9996 | 2 | 0.9999 | 1 | 1 | 2 | 0.9999 | 3 | 0.9977 | 2 | 1 | 1 | 1 | 3 | 0.9964 | 2 | 0.9649 |
| 29 |  |  | 2 | 0.9999 | 1 | 1 | 1 | 1 | 1 | 1 | 2 | 0.9999 | 3 | 0.9992 | 1 | 1 | 3 | 0.9974 | 1 | 1 |
| 30 | 1 | 1 | 1 | 1 | 1 | 1 | 1 | 1 | 1 | 1 | 1 | 1 | 2 | 0.9998 | 1 | 1 | 3 | 0.9999 | 1 | 1 |
| 31 | 2 | 0.9545 | 1 | 1 | 1 | 1 | 1 | 1 | 1 | 1 | 1 | 1 | 1 | 0.9999 | 1 | 1 | 1 | 1 | 1 | 1 |
