# Supplementary material for: Exhaustive Analysis of a Genotype Space Comprising 1015 Central Carbon Metabolisms Reveals an Organization Conducive to Metabolic Innovation
Source: PLoS Comput Biol. 2015 Aug 7;11(8):e1004329. doi: 10.1371/journal.pcbi.1004329 (PMC4529314; doi:10.1371/journal.pcbi.1004329)
Supplement: S3 Table — (DOCX) [file pcbi.1004329.s031.docx]

| Metabolism size | Acetate | Alphaketoglutarate | Fructose | Fumarate | Glucose | Glutamate | Lactate | Malate | Pyruvate | Succinate |
| --- | --- | --- | --- | --- | --- | --- | --- | --- | --- | --- |
| 23 |  |  | 100.0000 |  | 100.0000 |  |  |  |  |  |
| 24 |  |  | 96.0000 |  | 96.0000 |  |  |  |  |  |
| 25 |  | 100.0000 | 100.0000 |  | 100.0000 |  |  | 100.0000 |  |  |
| 26 |  | 50.0000 | 94.7700 | 100.0000 | 94.3200 | 75.0000 | 100.0000 | 100.0000 | 100.0000 |  |
| 27 |  | 37.1622 | 100.0000 | 100.0000 | 100.0000 | 60.7143 | 100.0000 | 86.2745 | 90.8696 | 100.0000 |
| 28 |  | 26.2136 | 90.9851 | 85.7143 | 92.1538 | 52.7132 | 100.0000 | 65.3465 | 73.3333 | 50.0000 |
| 29 |  | 15.6620 | 62.7200 | 50.0000 | 33.3800 | 43.8800 | 92.9167 | 43.5484 | 95.4071 | 75.0000 |
| 30 | 100.0000 | 16.9444 | 54.8400 | 39.8922 | 27.1270 | 34.7600 | 78.5294 | 45.9770 | 66.6667 | 50.0448 |
| 31 | 88.8889 | 4.0800 | 44.2900 | 28.6179 | 14.0625 | 24.9600 | 66.1900 | 33.8290 | 49.2063 | 35.9000 |
| 32 | 84.6154 | 9.4500 | 41.9130 | 20.5395 | 26.6667 | 1.8900 | 49.7417 | 37.5000 | 48.5000 | 27.6465 |
| 33 | 65.1556 | 13.9498 | 46.6667 | 36.7625 | 32.1970 | 6.4400 | 36.4474 | 44.1860 | 48.0769 | 37.5700 |
| 34 | 49.7000 | 27.0270 | 16.6667 | 32.6531 | 32.3800 | 15.4187 | 47.1338 | 36.5000 | 50.0000 | 30.9979 |
| 35 | 36.4300 | 38.1818 | 31.6279 | 37.8571 | 33.3334 | 18.9655 | 19.5652 | 30.7937 | 44.8276 | 19.2308 |
| 36 | 26.6200 | 35.4506 | 37.0900 | 38.3900 | 36.2900 | 29.6101 | 26.9231 | 39.3100 | 51.3766 | 37.5631 |
| 37 | 16.4163 | 40.5000 | 42.8900 | 43.7500 | 41.7100 | 35.1900 | 32.6600 | 44.2400 | 51.5900 | 41.2400 |
| 38 | 80.0000 | 45.3400 | 46.8500 | 49.5000 | 47.9400 | 41.3400 | 41.4900 | 50.7100 | 55.6800 | 46.6700 |
| 39 | 47.9769 | 57.1657 | 33.4000 | 32.5200 | 32.9300 | 31.9900 | 29.8100 | 34.9100 | 36.9600 | 30.7200 |
| 40 | 30.6100 | 43.4600 | 39.6600 | 39.7200 | 39.8400 | 39.6400 | 35.8700 | 42.1800 | 43.8800 | 37.9600 |
| 41 | 39.1100 | 50.3900 | 47.1400 | 49.0200 | 46.7000 | 47.5400 | 43.6200 | 50.6200 | 50.2900 | 46.4700 |
| 42 | 48.5400 | 58.2100 | 55.2300 | 56.4700 | 56.5700 | 55.6400 | 50.0600 | 58.1900 | 56.8300 | 54.7100 |
| 43 | 58.3500 | 65.7900 | 64.2900 | 64.5200 | 65.1800 | 63.0100 | 59.6800 | 66.4900 | 64.2400 | 61.1800 |
| 44 | 67.7000 | 74.0900 | 72.4300 | 72.0000 | 73.3800 | 72.4200 | 67.0500 | 74.1800 | 71.9800 | 70.9300 |
| 45 | 76.6500 | 80.7400 | 80.6200 | 80.1900 | 80.2300 | 78.4700 | 75.9600 | 80.4000 | 80.0800 | 77.8600 |
| 46 | 84.2100 | 88.9400 | 88.8300 | 88.3700 | 87.8100 | 87.9600 | 83.4800 | 88.9700 | 88.8900 | 84.6800 |
| 47 | 90.0700 | 91.2100 | 91.7800 | 91.1200 | 92.2700 | 90.9000 | 89.9000 | 92.2000 | 90.9500 | 90.6400 |
| 48 | 94.4000 | 95.2000 | 95.7700 | 94.8900 | 95.5700 | 95.1000 | 94.6100 | 95.8300 | 95.4600 | 94.4800 |
| 49 | 97.8800 | 98.0900 | 98.3100 | 98.0100 | 98.1900 | 97.8900 | 97.7700 | 98.1900 | 98.0800 | 97.5600 |
| 50 | 99.5074 | 99.5733 | 99.5951 | 99.5238 | 99.6154 | 99.5238 | 99.4958 | 99.5495 | 99.5733 | 99.4958 |
