# Supplementary material for: Exhaustive Analysis of a Genotype Space Comprising 1015 Central Carbon Metabolisms Reveals an Organization Conducive to Metabolic Innovation
Source: PLoS Comput Biol. 2015 Aug 7;11(8):e1004329. doi: 10.1371/journal.pcbi.1004329 (PMC4529314; doi:10.1371/journal.pcbi.1004329)
Supplement: S4 Table — (DOCX) [file pcbi.1004329.s032.docx]

| Metabolism size | Acetate | Alphaketoglutarate | Fructose | Fumarate | Glucose | Glutamate | Lactate | Malate | Pyruvate | Succinate |
| --- | --- | --- | --- | --- | --- | --- | --- | --- | --- | --- |
| 23 |  |  | 0 |  | 0 |  |  |  |  |  |
| 24 |  |  | 0 |  | 0 |  |  |  |  |  |
| 25 |  | 0 | 0 |  | 0 |  |  | 0 |  |  |
| 26 |  | 0 | 0 | 0 | 0 | 0 | 0 | 0 | 0 |  |
| 27 |  | 0 | 0 | 0 | 0 | 0 | 0 | 0 | 0 | 0 |
| 28 |  | 0 | 0 | 0 | 0 | 0 | 0 | 0 | 0 | 0 |
| 29 |  | 0 | 1 | 0 | 1 | 0 | 0 | 0 | 0 | 0 |
| 30 | 0 | 0 | 1 | 0 | 1 | 0 | 0 | 0 | 0 | 0 |
| 31 | 0 | 1 | 1 | 0 | 2 | 0 | 0 | 2 | 2 | 0 |
| 32 | 0 | 1 | 1 | 1 | 1 | 1 | 0 | 2 | 2 | 0 |
| 33 | 0 | 1 | 2 | 1 | 2 | 1 | 1 | 2 | 1 | 1 |
| 34 | 0 | 2 | 2 | 2 | 1 | 0 | 1 | 2 | 2 | 1 |
| 35 | 0 | 2 | 2 | 1 | 1 | 0 | 2 | 2 | 2 | 2 |
| 36 | 0 | 1 | 2 | 1 | 2 | 0 | 2 | 1 | 2 | 1 |
| 37 | 0 | 1 | 1 | 1 | 2 | 0 | 2 | 1 | 2 | 1 |
| 38 | 0 | 0 | 1 | 1 | 1 | 0 | 1 | 1 | 1 | 1 |
| 39 | 0 | 0 | 0 | 0 | 0 | 0 | 0 | 0 | 0 | 0 |
| 40 | 0 | 0 | 0 | 0 | 0 | 0 | 0 | 0 | 0 | 0 |
| 41 | 0 | 0 | 0 | 0 | 0 | 0 | 0 | 0 | 0 | 0 |
| 42 | 0 | 0 | 0 | 0 | 0 | 0 | 0 | 0 | 0 | 0 |
| 43 | 0 | 0 | 0 | 0 | 0 | 0 | 0 | 0 | 0 | 0 |
| 44 | 0 | 0 | 0 | 0 | 0 | 0 | 0 | 0 | 0 | 0 |
| 45 | 0 | 0 | 0 | 0 | 0 | 0 | 0 | 0 | 0 | 0 |
| 46 | 0 | 0 | 0 | 0 | 0 | 0 | 0 | 0 | 0 | 0 |
| 47 | 0 | 0 | 0 | 0 | 0 | 0 | 0 | 0 | 0 | 0 |
| 48 | 0 | 0 | 0 | 0 | 0 | 0 | 0 | 0 | 0 | 0 |
| 49 | 0 | 0 | 0 | 0 | 0 | 0 | 0 | 0 | 0 | 0 |
| 50 | 0 | 0 | 0 | 0 | 0 | 0 | 0 | 0 | 0 | 0 |
