# Supplementary material for: Exhaustive Analysis of a Genotype Space Comprising 1015 Central Carbon Metabolisms Reveals an Organization Conducive to Metabolic Innovation
Source: PLoS Comput Biol. 2015 Aug 7;11(8):e1004329. doi: 10.1371/journal.pcbi.1004329 (PMC4529314; doi:10.1371/journal.pcbi.1004329)
Supplement: S5 Table — The information in this table is restricted to metabolisms without disconnected reactions. (DOCX) [file pcbi.1004329.s033.docx]

| *n* | Acetate | | Alpha-ketoglutarate | | Fructose | | Fumarate | | Glucose | | Glutamate | | Lactate | | Malate | | Pyruvate | | Succinate | |
| --- | --- | --- | --- | --- | --- | --- | --- | --- | --- | --- | --- | --- | --- | --- | --- | --- | --- | --- | --- | --- |
|  | *n_C_* | *r_G_* | *n_C_* | *r_G_* | *n_C_* | *r_G_* | *n_C_* | *r_G_* | *n_C_* | *r_G_* | *n_C_* | *r_G_* | *n_C_* | *r_G_* | *n_C_* | *r_G_* | *n_C_* | *r_G_* | *n_C_* | *r_G_* |
| 23 |  |  |  |  | 2 | 0.6666 |  |  | 2 | 0.6666 |  |  |  |  |  |  |  |  |  |  |
| 24 |  |  |  |  | 2 | 0.6190 |  |  | 2 | 0.6190 |  |  |  |  |  |  |  |  |  |  |
| 25 |  |  |  | 1 | 2 | 0.9964 |  |  | 2 | 0.9965 |  |  |  |  | 1 | 1 |  |  |  |  |
| 26 |  |  | 2 | 0.9622 | 2 | 0.9907 | 1 | 1 | 2 | 0.9908 | 1 | 1 | 2 | 1 | 2 | 0.96 | 3 | 0.3333 |  |  |
| 27 |  |  | 2 | 0.9833 | 1 | 1 | 2 | 0.9583 | 1 | 1 | 3 | 0.9322 | 1 | 0.9574 | 1 | 0.9983 | 4 | 0.6564 | 1 | 1 |
| 28 |  |  | 3 | 0.9994 | 2 | 0.9999 | 1 | 0.9982 | 2 | 0.9999 | 3 | 0.9869 | 2 | 0.9962 | 1 | 1 | 3 | 0.7072 | 2 | 0.9607 |
| 29 |  |  | 2 | 0.9999 | 1 | 1 | 1 | 1 | 1 | 1 | 2 | 0.9998 | 3 | 0.9989 | 1 | 1 | 3 | 0.7352 | 1 | 0.9983 |
| 30 | 1 | 1 | 1 | 1 | 1 | 1 | 1 | 1 | 1 | 1 | 1 | 1 | 2 | 0.9989 | 1 | 1 | 3 | 0.7698 | 1 | 1 |
| 31 | 2 | 0.95 | 1 | 1 | 1 | 1 | 1 | 1 | 1 | 1 | 1 | 1 | 1 | 0.9998 | 1 | 1 | 1 | 0.8041 | 1 | 1 |
| 32 | 1 | 1 | 1 | 1 | 1 | 1 | 1 | 1 | 1 | 1 | 1 | 1 | 1 | 1 | 1 | 1 | 1 | 0.8386 | 1 | 1 |
| 33 | 1 | 1 | 1 | 1 | 1 | 1 | 1 | 1 | 1 | 1 | 1 | 1 | 1 | 1 | 1 | 1 | 1 | 0.8717 | 1 | 1 |
| 34 | 1 | 1 | 1 | 1 | 1 | 1 | 1 | 1 | 1 | 1 | 1 | 1 | 1 | 1 | 1 | 1 | 1 | 0.9016 | 1 | 1 |
| 35 | 1 | 1 | 1 | 1 | 1 | 1 | 1 | 1 | 1 | 1 | 1 | 1 | 1 | 1 | 1 | 1 | 1 | 0.9273 | 1 | 1 |
| 36 | 1 | 1 | 1 | 1 | 1 | 1 | 1 | 1 | 1 | 1 | 1 | 1 | 1 | 1 | 1 | 1 | 1 | 0.9480 | 1 | 1 |
| 37 | 1 | 1 | 1 | 1 | 1 | 1 | 1 | 1 | 1 | 1 | 1 | 1 | 1 | 1 | 1 | 1 | 1 | 0.9640 | 1 | 1 |
| 38 | 1 | 1 | 1 | 1 | 1 | 1 | 1 | 1 | 1 | 1 | 1 | 1 | 1 | 1 | 1 | 1 | 1 | 0.9758 | 1 | 1 |
| 39 | 1 | 1 | 1 | 1 | 1 | 1 | 1 | 1 | 1 | 1 | 1 | 1 | 1 | 1 | 1 | 1 | 1 | 0.9842 | 1 | 1 |
| 40 | 1 | 1 | 1 | 1 | 1 | 1 | 1 | 1 | 1 | 1 | 1 | 1 | 1 | 1 | 1 | 1 | 1 | 0.9901 | 1 | 1 |
| 41 | 1 | 1 | 1 | 1 | 1 | 1 | 1 | 1 | 1 | 1 | 1 | 1 | 1 | 1 | 1 | 1 | 1 | 0.9940 | 1 | 1 |
| 42 | 1 | 1 | 1 | 1 | 1 | 1 | 1 | 1 | 1 | 1 | 1 | 1 | 1 | 1 | 1 | 1 | 1 | 0.9965 | 1 | 1 |
| 43 | 1 | 1 | 1 | 1 | 1 | 1 | 1 | 1 | 1 | 1 | 1 | 1 | 1 | 1 | 1 | 1 | 1 | 0.9981 | 1 | 1 |
| 44 | 1 | 1 | 1 | 1 | 1 | 1 | 1 | 1 | 1 | 1 | 1 | 1 | 1 | 1 | 1 | 1 | 1 | 0.9990 | 1 | 1 |
| 45 | 1 | 1 | 1 | 1 | 1 | 1 | 1 | 1 | 1 | 1 | 1 | 1 | 1 | 1 | 1 | 1 | 1 | 0.9996 | 1 | 1 |
| 46 | 1 | 1 | 1 | 1 | 1 | 1 | 1 | 1 | 1 | 1 | 1 | 1 | 1 | 1 | 1 | 1 | 1 | 0.9998 | 1 | 1 |
| 47 | 1 | 1 | 1 | 1 | 1 | 1 | 1 | 1 | 1 | 1 | 1 | 1 | 1 | 1 | 1 | 1 | 1 | 0.9999 | 1 | 1 |
