# Supplementary material for: Exhaustive Analysis of a Genotype Space Comprising 1015 Central Carbon Metabolisms Reveals an Organization Conducive to Metabolic Innovation
Source: PLoS Comput Biol. 2015 Aug 7;11(8):e1004329. doi: 10.1371/journal.pcbi.1004329 (PMC4529314; doi:10.1371/journal.pcbi.1004329)
Supplement: S7 Table — (DOCX) [file pcbi.1004329.s035.docx]

|  | Number *k* of carbon sources | | | | | | | | | | | | | | | | | | | |
| --- | --- | --- | --- | --- | --- | --- | --- | --- | --- | --- | --- | --- | --- | --- | --- | --- | --- | --- | --- | --- |
| Number *n* of reactions | *k = 1* | | *k = 2* | | *k = 3* | | *k = 4* | | *k = 5* | | *k = 6* | | *k = 7* | | *k = 8* | | *k = 9* | | *k = 10* | |
|  | *n_C_* | *r_G_* | *n_C_* | *r_G_* | *n_C_* | *r_G_* | *n_C_* | *r_G_* | *n_C_* | *r_G_* | *n_C_* | *r_G_* | *n_C_* | *r_G_* | *n_C_* | *r_G_* | *n_C_* | *r_G_* | *n_C_* | *r_G_* |
| 23 | 2 | 0.6666 | 2 | 0.6666 |  |  |  |  |  |  |  |  |  |  |  |  |  |  |  |  |
| 24 | 2 | 0.6373 | 2 | 0.6373 |  |  |  |  |  |  |  |  |  |  |  |  |  |  |  |  |
| 25 | 2 | 0.9969 | 2 | 0.9969 |  |  |  |  |  |  |  |  |  |  |  |  |  |  |  |  |
| 26 | 3 | 0.992 | 2 | 1 |  |  |  |  |  |  |  |  |  |  |  |  |  |  |  |  |
| 27 | 4 | 1 | 2 | 1 | 1 | 1 |  |  |  |  |  |  |  |  |  |  |  |  |  |  |
| 28 | 3 | 1 | 4 | 1 | 4 | 1 | 1 | 1 |  |  |  |  |  |  |  |  |  |  |  |  |
| 29 | 3 | 0.9999 | 4 | 1 | 4 | 1 | 4 | 1 | 1 | 1 |  |  |  |  |  |  |  |  |  |  |
| 30 | 3 | 0.9999 | 5 | 1 | 5 | 1 | 5 | 1 | 5 | 1 | 1 | 1 | 1 | 1 |  |  |  |  |  |  |
| 31 | 2 | 0.9999 | 5 | 1 | 7 | 1 | 7 | 1 | 7 | 1 | 7 | 1 | 3 | 1 | 1 | 1 |  |  |  |  |
| 32 | 1 | 1 | 6 | 1 | 7 | 1 | 7 | 1 | 7 | 1 | 7 | 1 | 7 | 1 | 4 | 1 | 1 | 1 |  |  |
| 33 | 1 | 1 | 3 | 1 | 7 | 1 | 7 | 1 | 7 | 1 | 7 | 1 | 6 | 1 | 5 | 1 | 4 | 1 |  |  |
| 34 | 1 | 1 | 3 | 1 | 5 | 1 | 7 | 1 | 7 | 1 | 6 | 1 | 6 | 1 | 6 | 1 | 5 | 1 | 1 | 1 |
| 35 | 1 | 1 | 1 | 1 | 3 | 1 | 3 | 1 | 3 | 1 | 3 | 1 | 3 | 1 | 3 | 1 | 3 | 0.9995 | 3 | 0.9113 |
| 36 | 1 | 1 | 1 | 1 | 1 | 1 | 2 | 1 | 2 | 1 | 2 | 1 | 2 | 1 | 2 | 1 | 2 | 1 | 2 | 0.9959 |
