# Supplementary material for: Exhaustive Analysis of a Genotype Space Comprising 1015 Central Carbon Metabolisms Reveals an Organization Conducive to Metabolic Innovation
Source: PLoS Comput Biol. 2015 Aug 7;11(8):e1004329. doi: 10.1371/journal.pcbi.1004329 (PMC4529314; doi:10.1371/journal.pcbi.1004329)
Supplement: S8 Table — (DOCX) [file pcbi.1004329.s036.docx]

| Number *n* of reactions | *k = 1* | | *k = 2* | | *k = 3* | | *k = 4* | | *k = 5* | | *k = 6* | | *k = 7* | | *k = 8* | | *k = 9* | | *k = 10* | |
| --- | --- | --- | --- | --- | --- | --- | --- | --- | --- | --- | --- | --- | --- | --- | --- | --- | --- | --- | --- | --- |
|  | *n_C_* | *r_G_* | *n_C_* | *r_G_* | *n_C_* | *r_G_* | *n_C_* | *r_G_* | *n_C_* | *r_G_* | *n_C_* | *r_G_* | *n_C_* | *r_G_* | *n_C_* | *r_G_* | *n_C_* | *r_G_* | *n_C_* | *r_G_* |
| 23 | 2 | 0.6666 | 2 | 0.6666 |  |  |  |  |  |  |  |  |  |  |  |  |  |  |  |  |
| 24 | 2 | 0.6373 | 2 | 0.6373 |  |  |  |  |  |  |  |  |  |  |  |  |  |  |  |  |
| 25 | 2 | 0.9969 | 2 | 0.9969 |  |  |  |  |  |  |  |  |  |  |  |  |  |  |  |  |
| 26 | 2 | 0.3333 | 1 | 0.9919 |  |  |  |  |  |  |  |  |  |  |  |  |  |  |  |  |
| 27 | 1 | 0.9425 | 1 | 0.5 | 1 | 1 |  |  |  |  |  |  |  |  |  |  |  |  |  |  |
| 28 | 1 | 0.9649 | 1 | 0.2857 | 1 | 0.2857 | 1 | 1 |  |  |  |  |  |  |  |  |  |  |  |  |
| 29 | 2 | 0.9974 | 1 | 0.5049 | 1 | 0.2857 | 1 | 0.2857 | 1 | 1 |  |  |  |  |  |  |  |  |  |  |
| 30 | 3 | 0.9998 | 1 | 0.7142 | 1 | 0.3809 | 1 | 0.3809 | 1 | 0.3809 | 1 | 1 | 1 | 1 |  |  |  |  |  |  |
| 31 | 2 | 0.9545 | 1 | 0.7377 | 1 | 0.4 | 1 | 0.3809 | 1 | 0.3809 | 1 | 0.3809 | 1 | 0.6666 | 1 | 1 |  |  |  |  |
| 32 | 1 | 1 | 1 | 0.9518 | 1 | 0.4122 | 1 | 0.4122 | 1 | 0.4122 | 1 | 0.4122 | 1 | 0.6666 | 1 | 0.8256 | 1 | 1 |  |  |
| 33 | 1 | 1 | 1 | 0.9268 | 1 | 0.9232 | 1 | 0.6043 | 1 | 0.6043 | 1 | 0.6043 | 1 | 0.6043 | 1 | 0.8076 | 1 | 0.819 |  |  |
| 34 | 1 | 1 | 1 | 0.9975 | 1 | 0.9135 | 1 | 0.9135 | 1 | 0.9135 | 1 | 0.9135 | 1 | 0.9135 | 1 | 0.9135 | 1 | 0.9156 | 1 | 1 |
| 35 | 1 | 1 | 1 | 1 | 1 | 0.9961 | 1 | 0.909 | 1 | 0.909 | 1 | 0.909 | 1 | 0.909 | 1 | 0.909 | 2 | 0.909 | 3 | 0.9113 |
| 36 | 1 | 1 | 1 | 1 | 1 | 1 | 1 | 0.9957 | 1 | 0.9957 | 1 | 0.9957 | 1 | 0.9957 | 1 | 0.9957 | 1 | 0.9957 | 2 | 0.9959 |
